# The Translation Initiation Factor eIF4E Regulates the Sex-Specific Expression of the Master Switch Gene *Sxl* in *Drosophila melanogaster*

**DOI:** 10.1371/journal.pgen.1002185

**Published:** 2011-07-28

**Authors:** Patricia L. Graham, Judith L. Yanowitz, Jill K. M. Penn, Girish Deshpande, Paul Schedl

**Affiliations:** 1 Department of Molecular Biology, Princeton University, Princeton, New Jersey, United States of America; 2 Magee-Womens Research Institute, Pittsburgh, Pennsylvania, United States of America; University of California San Francisco, United States of America

## Abstract

In female fruit flies, *Sex-lethal* (*Sxl*) turns off the X chromosome dosage compensation system by a mechanism involving a combination of alternative splicing and translational repression of the *male specific lethal-2* (*msl-2*) mRNA. A genetic screen identified the translation initiation factor *eif4e* as a gene that acts together with *Sxl* to repress expression of the Msl-2 protein. However, *eif4e* is not required for *Sxl* mediated repression of *msl-2* mRNA translation. Instead, *eif4e* functions as a co-factor in *Sxl*-dependent female-specific alternative splicing of *msl-2* and also *Sxl* pre-mRNAs. Like other factors required for *Sxl* regulation of splicing, *eif4e* shows maternal-effect female-lethal interactions with *Sxl*. This female lethality can be enhanced by mutations in other co-factors that promote female-specific splicing and is caused by a failure to properly activate the *Sxl-*positive autoregulatory feedback loop in early embryos. In this feedback loop Sxl proteins promote their own synthesis by directing the female-specific alternative splicing of *Sxl-Pm* pre-mRNAs. Analysis of pre-mRNA splicing when *eif4e* activity is compromised demonstrates that *Sxl*-dependent female-specific splicing of both *Sxl-Pm* and *msl-2* pre-mRNAs requires *eif4e* activity. Consistent with a direct involvement in *Sxl*-dependent alternative splicing, eIF4E is associated with unspliced *Sxl-Pm* pre-mRNAs and is found in complexes that contain early acting splicing factors—the U1/U2 snRNP protein Sans-fils (Snf), the U1 snRNP protein U1-70k, U2AF38, U2AF50, and the Wilms' Tumor 1 Associated Protein Fl(2)d—that have been directly implicated in *Sxl* splicing regulation.

## Author Summary

Gene expression in eukaryotes is a complex process that occurs in several discrete steps. Some of those steps are separated into different sub-cellular compartments and thus might be expected to occur independently of one another and involve entirely distinct factors. For example pre-mRNA splicing takes place in the nucleus where it is coupled with transcription, while mRNA translation requires export to the cytoplasm and ribosome loading. We describe studies on the fruit fly *Drosophila* which indicate that a cytoplasmic translation initiation factor, the cap binding protein eIF4E, plays a key role in alternative splicing in the nucleus. When eIF4E activity is compromised, we observe defects in sex-specific splicing of pre-mRNAs that are regulated by the sex determination master switch gene *Sex-lethal*. Our data argue that eIF4E likely plays a direct role in the regulation of alternative splicing by *Sex-lethal*.

## Introduction

Translation initiation is mediated by the binding of a pre-initiation complex to the 5′ cap of the mRNA (reviewed in [1], [2]) that in turn recruits the small subunit of the 40S ribosome to the mRNA. The pre-initiation complex consists of the cap binding protein, eIF4E, and a scaffolding protein, eIF4G, which mediates interactions with various components of the 40S initiation complex. In many organisms there is also a third protein in the complex, eIF4A, an ATP dependent RNA helicase. Modulating eIF4E activity appears to be a key control point for regulating translation. One of the most common mechanisms of regulation is by controlling the association eIF4E with eIF4G. Factors such as poly-A binding protein that promote the association between eIF4E and eIF4G activate translation initiation, while factors such as the 4E-binding proteins (4E-BPs) that block their association, inhibit initiation [3], [4].

Although eIF4E's primary function in the cell is in regulating translation initiation, studies over the past decade have revealed unexpected activities for eIF4E at steps prior to translation. Among the more surprising findings is that there are substantial amounts of eIF4E in eukaryotic nuclei [5]–[9]. One role for eIF4E in the nucleus is the transport of specific mRNAs, like cyclin D1, to the cytoplasm [10]. This eIF4E activity is distinct from translation initiation since an eIF4E mutation that prevents it from forming an active translation complex still allows cyclin D1 mRNA transport [8]. The transport function of eIF4E is modulated by at least two other proteins, PML and PRH [11], [12]. While PML seems to be ubiquitously expressed, PRH is found only in specific tissues [13]. In addition, the intracellular distribution of eIF4E exhibits dynamic changes during *Xenopus* development [9]. These observation raise the possibility that eIF4E might have additional functions in the nucleus during development. Consistent with this idea, we show here that eIF4E plays a novel role in the process of sex determination in *Drosophila melanogaster*.

**Figure 1:**
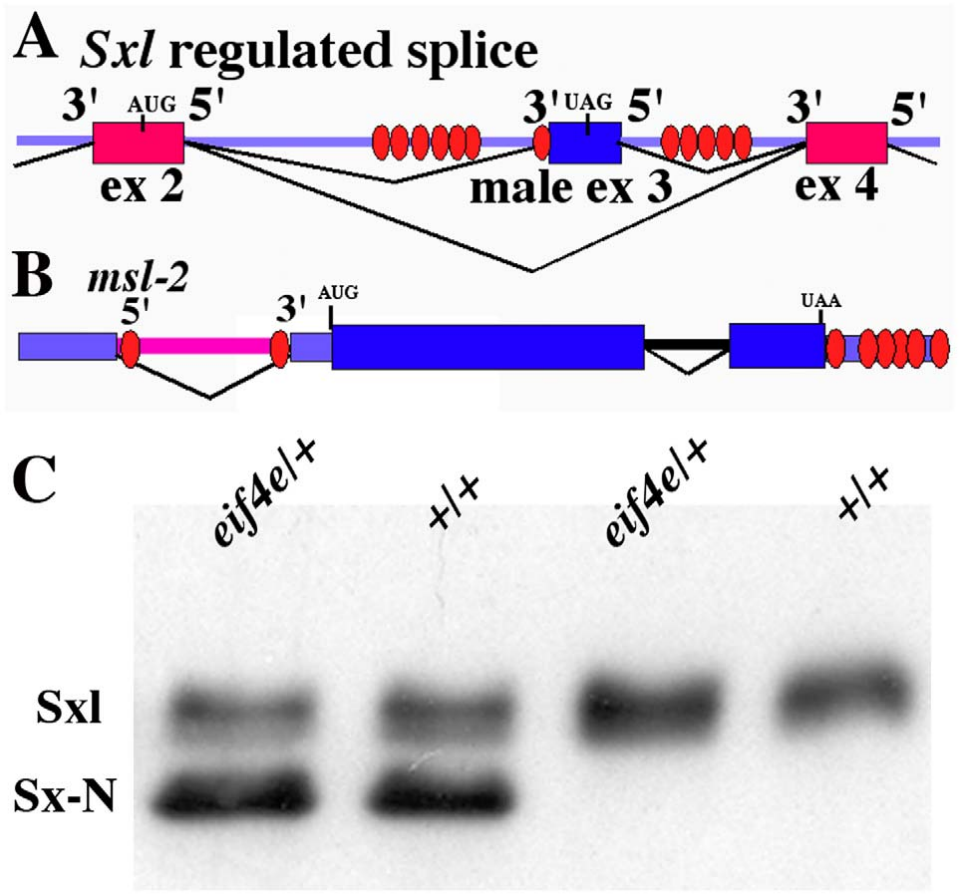
Sx-N protein can repress the translation of endogenous *Sxl-Pm* mRNAs in an *eif4e* mutant background. A) Model of the alternatively spliced region of *Sxl* (exons 2, 3 and 4). Sxl binding sites are shown as ovals. In males exon2 (ex2) is joined to exon3 (ex3) which is in turn joined to exon4 (ex4). The stop codon within exon 3 causes male transcripts to produce a truncated protein. In females Sxl protein prevents inclusion of exon3, and exon2 is joined directly to exon4. B) Model of the *msl-2* gene. The Sxl binding sites are shown as ovals. In males the intron in the 5′UTR that contains the two Sxl sites is spliced out by the default splicing machinery. In females Sxl protein blocks the splicing of the 5′UTR intron and the two Sxl sites in the intron are retained. Binding of Sxl to these two sites and sites in the 3′UTR represses translation of *msl-2* mRNA. C) Western blot of Sxl proteins from *eif4e/+ hsp83:Sx-NΔ* transgene females (lane 1), *+/+ hsp83:Sx-NΔ* transgene females (lane 2), *eif4e/+* (lane 3) and *+/+* (lane 4) females. The presence or absence of the *eif4e* mutation is indicated above each lane. Levels of both Sx-N protein and endogenous Sxl protein are unaffected by the presence of the *eif4e* mutation.

Sex determination in the fly is controlled by the master regulatory switch gene *Sex-lethal* (*Sxl*) (reviewed in [14]–[16]). The activity state of the *Sxl* gene is selected early in development by an X chromosome counting system. The target for the X/A signaling system is the *Sxl* establishment promoter, *Sxl-Pe* [17]. When there are two X chromosomes, *Sxl-Pe* is turned on, while it remains off when there is a single X chromosome. *Sxl-Pe* mRNAs encode RRM type RNA binding proteins which mediate the transition from the initiation to the maintenance mode of *Sxl* regulation by directing the female-specific splicing of the first pre-mRNAs produced from a second, upstream promoter, the maintenance promoter, *Sxl-Pm* [18], [19]. *Sxl-Pm* is turned on before the blastoderm cellularizes, just as *Sxl-Pe* is being shut off. In the presence of Sxl-Pe proteins, the first *Sxl-Pm* transcripts are spliced in the female-specific pattern in which exon 2 is joined to exon 4 (see Figure 1A). The resulting *Sxl-Pm* mRNAs encode Sxl proteins that direct the female specific splicing of new *Sxl-Pm* pre-mRNAs and this establishes a positive autoregulatory feedback loop that maintains the *Sxl* gene in the “on” state for the remainder of development. In male embryos, which lack the Sxl-Pe proteins, the *Sxl-Pm* pre-mRNAs are spliced in the default pattern, incorporating the male specific exon 3 (Figure 1A). This exon has several in-frame stop codons that prematurely truncate the open reading frame so that male specific *Sxl-Pm* mRNAs produce only small non-functional polypeptides. As a consequence the *Sxl* gene remains off throughout development in males.

In females, *Sxl* orchestrates sexual development by regulating the alternative splicing of *transformer* (*tra*) pre-mRNAs [20]–[23]. Like *Sxl*, functional Tra protein is only produced by female-specific *tra* mRNAs, while mRNAs spliced in the default, male pattern encode non-functional polypeptides. *Sxl* also negatively regulates the dosage compensation system, which is responsible for hyperactivating X-linked transcription in males, by repressing *male-specific lethal-2* (*msl-2*). *Sxl* represses *msl-2* by first blocking the splicing of an intron in the 5′ UTR of the *msl-2* pre-mRNA (see Figure 1B), and then by inhibiting the translation of the mature mRNA [24]–[31]. In addition, there are two other known targets for *Sxl* translational repression. One is the *Sxl* mRNA itself. Sxl binds to target sequences in the *Sxl* 5′ and 3′ UTRs and downregulates translation. It is thought that this negative autoregulatory activity provides a critical homeostasis mechanism that prevents the accumulation of excess Sxl protein. This is important as too much Sxl can disrupt development and have female lethal effects [32]. The other known target is the *Notch* (*N*) mRNA [33]. *Sxl*-dependent repression of *N* mRNA translation is important for the elaboration of sexually dimorphic traits in females. Like *msl-2* and *Sxl*, translational repression appears to be mediated by Sxl binding to sites in the *N* UTRs.

Translational repression of *msl-2* mRNA by Sxl is thought to involve two separate mechanisms acting coordinately. Binding sites for Sxl in the unspliced intron in the 5′ UTR and in the 3′UTR of *msl-*2 are required for complete repression [25], [26]. Sxl binding to the 5′UTR blocks recruitment of the 40S pre-initiation complex [31], [34]. While factors that act with Sxl at the 5′UTR of *msl-2* have yet to be identified, repression by the 3′UTR requires Sxl, PABP and a co-repressor UNR [35]–[37]. Somewhat unexpectedly, this complex does not affect recruitment of eIF4E or eIF4G to the 5′ end. Instead it prevents ribosomes that do manage to attach to the *msl-2* mRNA from scanning [31], [38].

Although eIF4E does not appear to be a key player in the translational repression of *msl-2* mRNAs, we report here that it has an important role in the process of sex determination in *Drosophila*. We find that eIF4E activity is required in females to stably activate and maintain the *Sxl* positive autoregulatory feedback loop and to efficiently repress *msl-2*. Surprisingly, this requirement for eIF4E activity in fly sex determination is in promoting the female-specific splicing of the *Sxl* and *msl-2* transcripts, not in translational regulation.

## Results

### Mutations in *eif4e* rescue males expressing a *Sxl* transgene

In previous studies we examined the biological properties of a truncated Sxl protein, Sx-N, that contains both RRM RNA binding domains, but is missing 40 amino acids from the N-terminus [39]. We found that the splicing activity of Sx-N is impaired; it can not direct the female-specific splicing of *tra* and has substantially reduced autoregulatory activity. However, the truncated protein is able to inhibit the translation of *msl-2* mRNA and kills males even in the absence of a wild type *Sxl* gene. As would be expected if the male lethal effects of Sx-N are due to repression of *msl-2* mRNA translation, *hsp83:Sx-NΔ* males can be fully rescued by an *hsp83:msl-2* transgene that lacks the Sxl binding sites in the 5′ and 3′ UTRs.

With the aim of discovering factors important for Sxl dependent repression of *msl-2* we screened for deletions that dominantly suppress the male lethal effects (in a *Sxl* ^−^ background) of a transgene, *hsp83:Sx-NΔ*, that constitutively expresses the truncated Sx-N protein. We then identified the interacting locus by testing mutations mapping to the suppressing deletion. We anticipated that genes recovered in this screen would fall into two general classes. In the first would be genes required for efficient expression of Sx-N by the transgene. Consistent with this expectation, one of the suppressing mutations was the heat shock factor, *hsf*. Genes in the second class would be required for efficient repression of *msl-2* by the truncated Sx-N protein. In this group we expected to find factors required by Sxl to inhibit *msl-2* translation; however, since the Sxl binding sites in the *msl-2* 5′ UTR intron are needed to completely repress translation, we anticipated that we might also recover genes that collaborate with *Sxl* to block the removal of this intron [25], [26], [28], [31].

One of the candidate translation factors recovered in the screen was the *eif4e* gene, which encodes the cap binding protein. Three independent alleles of *eif4e* were tested. In an otherwise wild type background less than one in 10^3^ *Sxl* ^−^ males carrying the *hsp83:Sx-NΔ* transgene survive. By contrast, when the *hsp83:Sx-NΔ; Sxl* ^−^ males were also heterozygous for an *eif4e* mutation, between 2% and 9% of the transgenic males survived depending upon the allele.

### *eif4e* mutations do not impair the negative autoregulatory activity of the Sx-N protein

Since Sxl-dependent repression of *msl-2* translation *in vitro* is independent of the cap and does not seem to be mediated through interactions with eIF4E [34], [38], it was surprising that *eif4e* was recovered in our screen. However, it seemed possible that an *in vivo* requirement for *eif4e* activity might be bypassed in *in vitro* translation systems. In this case, the levels of Msl-2 should increase in *hsp83:Sx-NΔ* transgene males when they are heterozygous for one of the *eif4e* mutations. However, testing whether *eif4e* mutations perturb Sx-N dependent translational repression of *msl-2* mRNA in adults or at earlier stages of development is complicated by the male-lethal effects of the truncated Sxl protein.

To circumvent this complication, we tested the effects *eif4e* on *Sxl* negative autoregulation as this can be done in females where Sx-N doesn't have such deleterious consequences. The endogenous *Sxl-Pm* mRNAs have one Sxl binding site in the 5′ UTR, while there can be eight or more in the 3′ UTR. Sxl binds to these sites and downregulates translation. Though the truncated Sx-N protein can also repress translation of *Sxl-Pm* mRNAs, its inhibitory effects are somewhat weaker than the full-length protein [39]. However, it is possible to detect Sx-N repression of endogenous *Sxl* mRNAs using the *hsp83:Sx-NΔ* transgene. This transgene expresses *Sxl* mRNAs that lack the 5′ Sxl binding site and most of the 3′ UTR binding sites, and as a consequence are less sensitive to repression than the endogenous mRNAs [39]. For this reason, Sx-N protein produced by the transgene preferentially represses translation of the endogenous mRNAs and in *hsp83:Sx-NΔ* transgenic females the amount of Sx-N is typically greater than the two major endogenous Sxl proteins.

We compared the repression of the endogenous Sxl in *hsp83:Sx-NΔ* transgene females either wild type or heterozygous for *eif4e*. Figure 1C shows that in transgenic, wild type females the level of endogenous Sxl is less than Sx-N. Consistent with the results of the *in vitro* translation experiments, reducing *eif4e* activity does not have an obvious effect on repression of *Sxl-Pm* mRNAs by Sx-N and the ratio of the endogenous protein to Sx-N in *eif4e/+* females remains similar to that in wild type females. With the caveat that Sxl may require a different set of accessory proteins to repress the translation of each of its target mRNAs, this finding does not support the idea that eIF4E functions as a co-factor in Sxl inhibition of *msl-2* translation *in vivo*.

### *msl-2* mRNA splicing in *eif4e/+ hsp83:Sx-NΔ* transgene males

The alternative possibility is that *eif4e* rescues the male lethal effects of Sx-N because Sxl requires *eif4e* activity to effectively prevent the splicing of the intron in the 5′ UTR of *msl-2* pre-RNA. To test this idea, we examined the splicing pattern of *msl-2* mRNA in three surviving *Sxl^-^;eif4e/+; hsp83:Sx-NΔ* males. In wild type females, Sxl efficiently blocks the splicing of the *msl-2* 5′ UTR intron and in most female mRNAs the intron is unspliced. In wild type males the 5′ intron is spliced out of most *msl-2* mRNAs. As expected, we found that ectopically expressed Sx-N protein blocks the splicing of the 5′ intron and as shown for one of the surviving *Sxl* ^−^ *;eif4e/+; hsp83:Sx-NΔ* males in Figure S1, *msl-2* mRNA spliced in the female pattern is readily detected. However, we found that Sx-N wasn't able to fully inhibit the splicing of the 5′ intron, and roughly similar quantities of male spliced *msl-2* mRNAs were also observed (Figure S1). Equivalent levels of male spliced *msl-2* mRNAs were also found in both of the other *Sxl* ^−^ *;eif4e/+; hsp83:Sx-NΔ* males. Since the Sxl binding sites in the 5′ UTR are essential for efficient translational repression, Sx-N would not be able to completely block the translation of these male spliced *msl-2* mRNAs.

### *eif4e* is required for the stable activation of the *Sxl* positive autoregulatory feedback loop in early embryos

Though the results described in the previous section could explain why a small percentage of *eif4e/+* males escape the lethal effects of *Sx-NΔ*, it is not possible to determine if the relative amount of male spliced *msl-2* mRNA is increased compared to *eif4e^+^* males because the controls don't survive. However, as it seemed possible that the effects of *eif4e* on *Sxl* dependent splicing might not be limited to *msl-2*, we took advantage of a simple genetic test for genes involved in *Sxl* positive autoregulation. The initial activation of the positive *Sxl* autoregulatory loop in female embryos is sensitive to alterations in the dose of gene products that play a critical role in promoting the female specific splicing of *Sxl-Pm* pre-mRNAs. Because of this sensitivity, mutations in splicing factors like the U1A/U2B” snRNP protein Snf often show dominant female lethal interactions with *Sxl* [40]–[46].

If *eif4e* is required for female specific splicing, then dominant female lethal interactions with *Sxl* might be observed. In contrast, if *eif4e* is needed to help repress the translation of *Sxl* target mRNAs, then reducing *eif4e* activity should increase the translation of *Sxl* mRNAs and would be expected to suppress rather than enhance any female specific lethality. The results in Table 1 show that the former prediction is correct. All three of the *eif4e* alleles we tested, *eif4e^568^*, *eif4e^587/11^*, and *eif4e^715^*, showed dominant female lethal interactions with the null mutation *Sxl^f1^* (Table 1) [47]. These *eif4e* alleles are P-element insertions and are thought to be hypomorphic mutations [48]–[49]. The weakest allele, *eif4e^568^*, reduces female viability by a quarter, while female viability is reduced by a third to nearly a half for the two stronger alleles *eif4e^587/11^* and *eif4e^715^*. Although the reductions in female viability seen for the three *eif4e* mutations are not as great as that observed for the *snf* null allele *J210* or the dominant negative allele *1621*, they are roughly equivalent to that seen for the hypomorphic allele *JA2* (Table 1).

**Table 1:** *eIF4e* and *snf* interactions with *Sxl.*

Maternal Genotype	Female Viability x *Sxl^f1^*
*w*	98
	
*snf^JA2^/w*	69
*snf^J210^/w*	30
*snf^1621^/w*	20
	
*eif4e^568^*	75
*eif4e^587/11^*	66
*eif4e^715^*	54
	
*snf^1621^/w:eif4e^568^/+*	10
*snf^1621^/w:eif4e^587/11^/+*	2

Maternal Genotype	Female Viability x *Sxl^7BO^*
*snf^1621^/w*	14
	
*eif4e^587/11^*	61
*eif4e^715^*	34
	
*snf^1621^/w:eif4e^587/11^/+*	0.3

Maternal Genotype	Female Viability x *Sxl^f9^*
*w*	112
*eif4e^587/11^*	99
*eif4e^715^*	84

Females heterozygous for the indicated mutation(s) were crossed to *Sxl^f1^*, *Sxl^7BO^* or *Sxl^f9^* males at 29°C. Female viability was calculated as ((#females)/(#males))100 except in crosses with *snf* mutations that affected male viability. In those crosses female viability was calculated as ((# females)/(2(non-mutant males))100. Except w, a minimum of 700 progeny were scored for each cross.

In the experiments described above the *eif4e/+* females were crossed to *Sxl^f1^* males giving two classes of *Sxl^f1^* progeny, those carrying the e*if4e* mutation and those with the wild type chromosome. We noticed that the viability of both classes of *Sxl^f1^* progeny were affected equally (data not shown) suggesting that the lethality is predominantly the result of a lowered maternal contribution of eIF4E rather than a reduction in zygotic eIF4E. Consistent with this conclusion, when we did the reciprocal cross in which the *eif4e* mutation was introduced from the father and the *Sxl* mutation introduced from the mother, we found that the viability of *Sxl* ^−^ */+* females was close to that of wild type females (not shown).

To confirm that the female lethal interactions are due to a reduction in *eif4e* activity, we tested whether they can be rescued by an *eif4e* transgene. Two isoforms of eIF4E are expressed *Drosophila*. We introduced transgenes expressing each isoform into *eif4e^715^/+* females and mated them to *Sxl^f1^* males. We found that both could suppress the maternal effect lethal interactions between *eif4E* and *Sxl* (data not shown). We also tested a second independent *Sxl* allele, *Sxl^7B0^* [50]. Like *Sxl^f1^*, *Sxl^7B0^* exhibited dominant female lethal interactions with *eif4e* (Table 1).

### *eif4e* mutations do not show dominant female lethal interactions with a mutation, *Sxl^f9^*, that only eliminates *Sxl-Pe* activity

The null mutations *Sxl^f1^* and *Sxl^7B0^* discussed above eliminate both early *Sxl* initiation functions provided by *Sxl-Pe* mRNAs and late *Sxl* sex determination functions (maintenance, sexual differentiation, and dosage compensation) provided by the *Sxl-Pm* mRNAs [47], [50]. While there are no known mutations that specifically eliminate only the late *Sxl* functions, the *Sxl^f9^* mutation disrupts the initiation function of the *Sxl-Pe* transcripts [51]–[52]. If the reduction in *eif4e* activity impairs the female-specific splicing of *Sxl-Pm* pre-mRNAs, then *eif4e* mutations should have a smaller effect on the viability of flies carrying a *Sxl* mutation that only affects the *Sxl-Pe* pre-mRNAs as these transcripts do not require Sxl for proper splicing [53]–[54]. As can be seen in Table 1, *Sxl^f9^* differs from *Sxl^f1^* and *Sxl^7B0^* in that it shows only a weak female lethal interaction with *eif4e* mutations. It also interacts much less strongly with *snf^1621^* than either of the *Sxl* null alleles (data not shown).

### Sxl protein expression is disrupted in progeny of *snf* and *eif4e* mothers

The female lethal interactions between *Sxl* and co-factors like *snf* that are critical for the female splicing of *Sxl-Pm* pre-mRNAs arise because the positive autoregulatory feedback loop is not properly set in motion [43]–[45]. However, there are no special requirements for these co- factors in the activation of *Sxl-Pe* by the X chromosome counting system or the splicing and translation of *Sxl-Pe* transcripts [53]–[54]. For these reasons, defects in Sxl accumulation are not observed in blastoderm stage embryos compromised for a sex-specific splicing co-factor. However, later in development, when protein expression depends upon female spliced *Sxl-Pm* mRNAs, the pattern of Sxl accumulation becomes abnormal. To determine if this is true for *eif4e* as well, we examined the expression of Sxl in blastoderm and post-blastoderm stage embryos.

**Figure 2:**
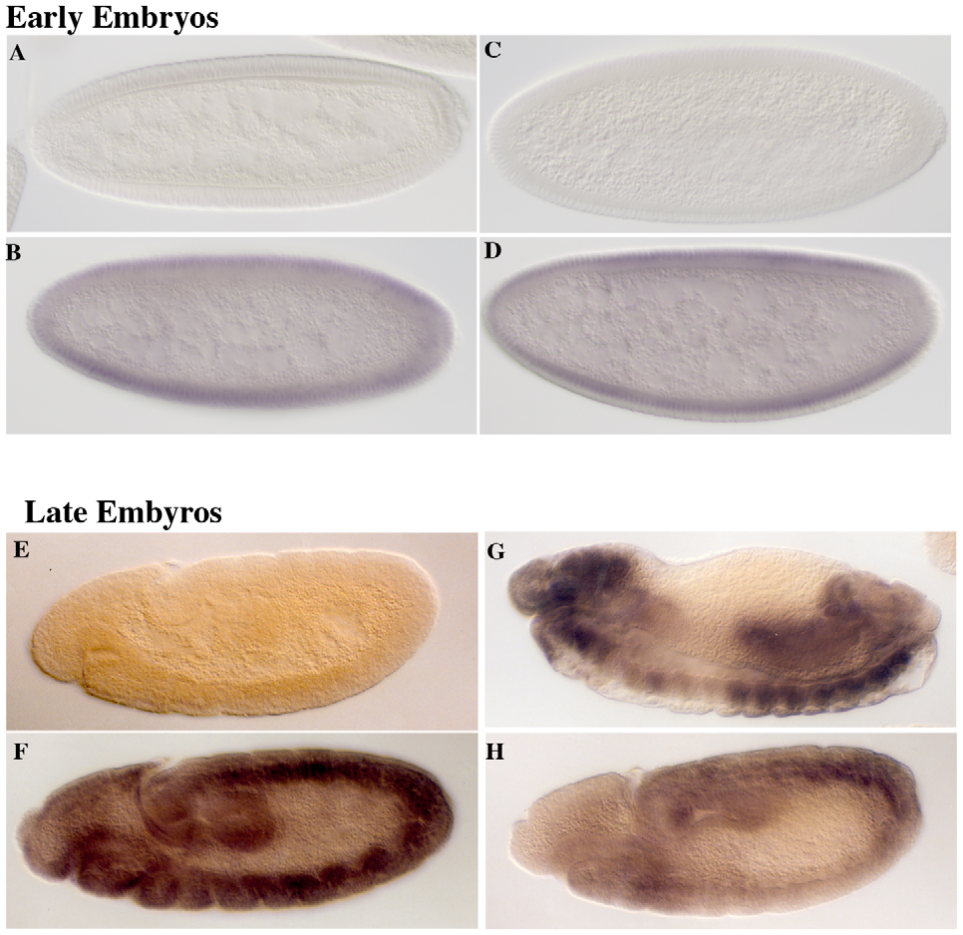
*eif4e* mutations alter expression of *Sxl* from the late, but not the early, promoter. Embryos from wild type (A, B, E, F) and *eif4e/+* (C, D, G, H) mothers crossed to *Sxl^f1^/Y* fathers were stained with antibody to Sxl. Male embryos from either cross do not express Sxl protein (A, C, E). Female embryos from wild type mothers express Sxl evenly throughout the embryo both early (B) and late (F). Female embryos from *eif4e/+* mothers express Sxl normally early (D), but often display patchy expression late (G,H).

Consistent with the idea that *eif4e* functions downstream of *Sxl-Pe*, *eif4e* mutations have no apparent effect on the expression of Sxl from the *Sxl-Pe* mRNAs. As shown in Figure 2 and Table S1, blastoderm stage progeny from *eif4e* ^−^ */+* and *snf* ^−^ */+* mothers crossed to *Sxl* ^−*f1*^ fathers resemble wild type in that about 50% of the embryos (females) express Sxl protein (compare panels A & B with C & D). While reducing *eif4e* activity does not perturb activation of *Sxl* by the X chromosome counting system, it does have a significant effect on the expression of Sxl in older embryos. In the wild type controls (either *w x w* or *w x Sxl^f1^),* high uniform levels of Sxl protein are observed in about 50% of the embryos, while a equal number show no staining (panels E & F). For the dominant negative *snf^1621^* allele only 11% of the embryos show the expected high uniform level of Sxl while Sxl expression in the remaining female embryos is either irregular or quite low (Table S1). As would be expected from the relative severity of the synthetic lethal interactions, the effects of the hypomorphic *eif4e* alleles on Sxl expression in post-cellular blastoderm embryos are not as strong as *snf^1621^*. For both *eif4e^587/11^* and *eif4e^713^* about one third of the embryos (or about two thirds of the females) show a high uniform level of Sxl accumulation (Table S1). The remaining female embryos show either a patchy pattern of Sxl protein accumulation or only low levels of protein (Figure 2G and 2H). These defects in Sxl expression in post-blastoderm embryos indicate that the *Sxl* autoregulatory feedback loop is not properly established in the female progeny of *eif4e* ^−^ */+* mothers.

### The constitutively active *Sxl^M^* mutations suppress the dominant female lethal interactions between *eif4e* and *Sxl*

To confirm that the female lethal effects of *eif4e* are due to a failure to activate the *Sxl* positive autoregulatory loop we tested whether *Sxl* ^−^ */+* female progeny of *eif4e* ^−^ */+*mothers can be rescued by three different gain-of-function *Sxl* alleles, *Sxl^M1^*, *Sxl^M4^*, and *Sxl^M6^*, that constitutively splice *Sxl-Pm* transcripts in the female mode [55]. As a positive control we generated an equivalent combination of *Sxl^M1^* and *snf^1621^*. Females *trans*-heterozygous for each combination were mated with *Sxl^f1^* males. As can be seen in Table S2 for the positive control, *Sxl^M1^* suppresses the maternal effect female lethal interactions between *snf* and *Sxl^f1^*. Similarly, *Sxl^M1^* and both of the other gain-of-function alleles also suppress the maternal effect lethal interactions between *eif4e^587/11^* and *Sxl^fl^*. In these crosses only half of the female progeny inherit the *Sxl* gain-of-function allele. As expected, most of the surviving females are the ones that carry the gain-of-function allele.

### Female embryos from *eif4e* ^−^ */+* mothers produce male *Sxl* transcripts

**Figure 3:**
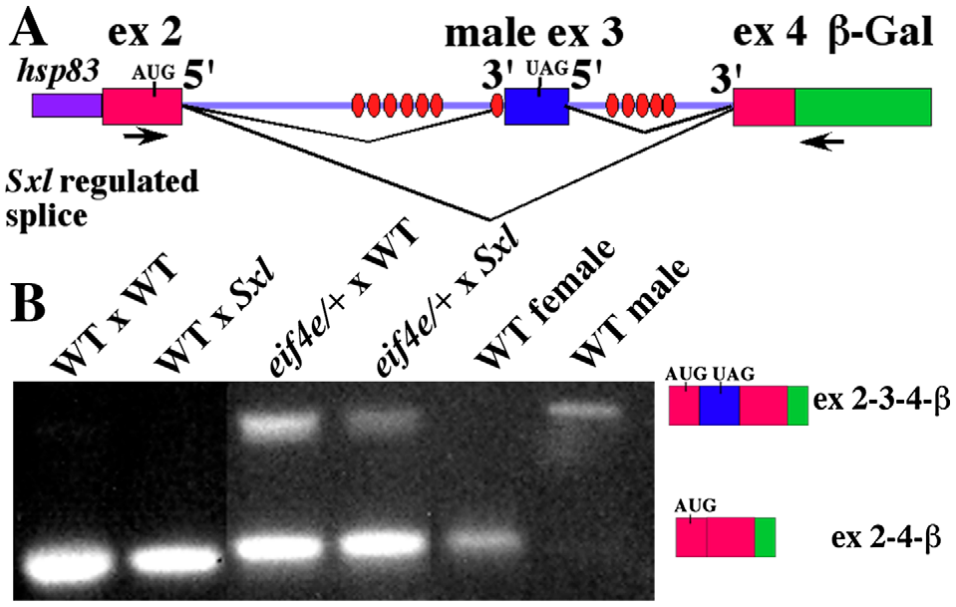
Female progeny of *eif4e/+* mothers produce male transcripts during early embryogenesis and in splicing compromised backgrounds. A) Model of the *Sxl* splicing reporter. Sxl binding sites are shown as ovals. Primers for PCR are indicated as arrows below the gene model. As indicated next to the gel, the female splice pattern skips exon3 (lane 5), while the male splice pattern includes exon3 (lane 6). B) RT-PCR was performed to analyze the products of an X-linked *Sxl* splice reporter brought from the male parent. Results were visualized with ethidium bromide. Female blastoderm stage embryos from wild type females express only female transcripts even when heterozygous for the *Sxl^f1^* mutation (lane 1, 2). Female blastoderm stage embryos from *eif4e/+* females express both the male and female transcripts (lane 3, 4).

If the positive autoregulatory loop is not properly activated when *eif4e* is compromised, we would expect to find male spliced *Sxl* transcripts in female blastoderm/early gastrula embryos. To examine the splicing pattern of *Sxl-Pm* transcripts specifically in female embryos during this period we took advantage of an X-linked *Sxl-Pm* splicing reporter. The splicing reporter has a *Sxl* genomic fragment extending across the regulated splice sites from exon 2 to exon 4 while exon 4 is fused to β-galactosidase sequences (see Figure 3A: [56]). Expression of the fusion gene is driven by the *hsp83* promoter. This promoter is activated in the zygote during the late syncytial blastoderm stage around the time when *Sxl-Pm* transcription commences [57]. Figure 3B shows that the transcripts spanning the regulated *Sxl* exon2-exon3-exon4 splicing cassette are spliced in the appropriate sex-specific pattern in control adult flies collected from a stock homozygous for the transgene: exon 2–4 in females and exons 2–3–4 in male.

Sxl*^f1^* or *Sxl^+^* males carrying the splicing reporter were crossed to *eif4e^587/11^/+* or control wild type females. To visualize the splicing of the regulated exon2-exon3-exon4 cassette when the autoregulatory feedback loop is first activated, we isolated RNA from 1–3 hr embryos and analyzed the structure of the transcripts expressed from the reporter by RT-PCR. When the mother is wild type we find that transcripts spanning the exon2-exon3-exon4 cassette are spliced exclusively in the female pattern (Figure 3B). This is true not only for female embryos that have two wild type copies of *Sxl* (fathers are *Sxl+/Y*), but also for female embryos that are heterozygous for the *Sxl^fl^* mutation (fathers are *Sxl^f1^/Y*). A different result is obtained when the mother is heterozygous for *eif4e^587/11^* (Figure 3B). In this case, we detect not only female but also male spliced reporter RNAs. With this allele, male spliced RNAs are observed in both *Sxl^fl^/+* embryos and in embryos that are wild type for *Sxl*. Similar results were obtained for *snf^1621^* (not shown). We also observed male spliced reporter RNAs in the female progeny of mothers heterozygous for two other *eif4e* alleles. However, for both of these *eif4e* alleles the male transcripts were only present when the female embryos were heterozygous for the *Sxl* mutation (not shown).

### Does eIF4E function in *Sxl*-dependent splicing regulation?

Two general mechanisms, one direct and the other indirect, could potentially account for the effects of *eif4e* on *Sxl* activation. In the direct mechanism, *eif4e* would function as a Sxl co-factor in the female specific processing of *Sxl-Pm* pre-mRNAs. In this case, reducing *eif4e* activity would compromise the female specific splicing of *Sxl-Pm* pre-mRNAs and prevent full activation of the positive autoregulatory feedback loop when the loop is first being initiated. In the second, *eif4e* would be required at a point subsequent to the splicing of the *Sxl-Pm* pre-mRNAs. For example, it may be needed in the cytoplasm for the efficient translation of *Sxl-Pm* mRNAs, or it might function in their nuclear export. In this scenario, the expression of Sxl proteins from the newly synthesized *Sxl-Pm* mRNAs would be impaired and sub-optimal levels of Sxl-Pm proteins would be produced. As a consequence, when the Sxl-Pe proteins decay, there would be an insufficient amount of Sxl remaining to stably maintain the positive autoregulatory feedback loop, and splicing would gradually switch from the female to the male pattern. Though our experiments with the splicing reporter suggest an immediate rather than a gradual effect on splicing of the *Sxl-Pm* transcripts, we cannot rule out the possibility that there is some disruption in the export or translation of *Sxl-Pm* mRNAs during the initial activation of the positive autoregulatory feedback loop. Moreover, consistent with the possible importance of post-splicing steps in *Sxl* activation, Stitzinger et al [58] found female lethal interactions with *Sxl* when mothers are simultaneously heterozygous for mutations in *aspartyl tRNA synthetase* and *snf*. Although the *aspartyl tRNA synthetase* mutants differ from *eif4e* in that they do not show female lethal interactions with *Sxl* on their own, the fact that reductions in the maternal dose of this synthetase can affect the activation of the autoregulatory loop lends credence to a post-splicing function. For these reasons we sought experimental paradigms in which we could assay for *eif4e* induced perturbations in *Sxl* dependent female-specific splicing under conditions in which the autoregulatory loop had already been “fully” activated and Sxl proteins were present at wild type levels.

### Effects of *eif4e* mutations on Sxl pre-mRNA splicing in a sensitized background

**Figure 4:**
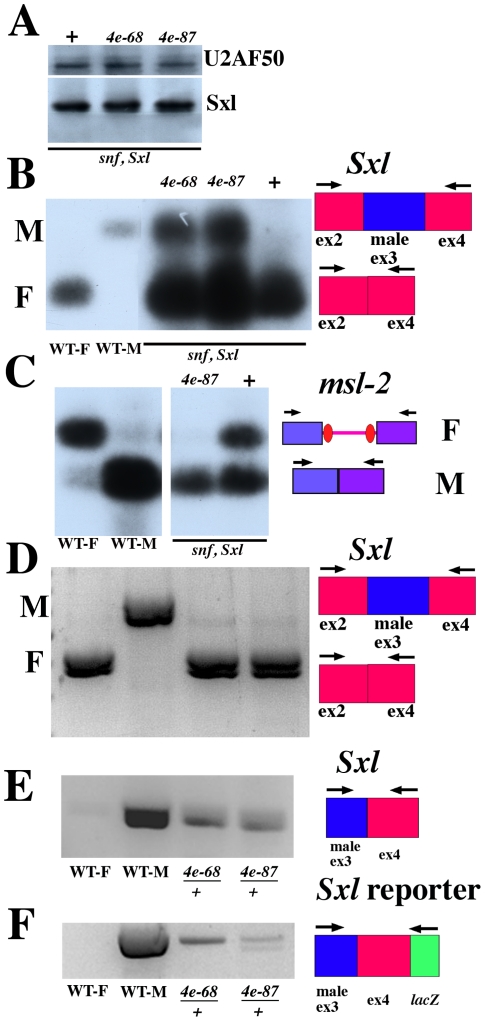
*eif4e* mutations shift Sxl regulated splicing toward male mode although Sxl protein levels are normal. A) Western blot of control *snf^1621^Sxl^f1^/++* (lane 1) females and of *snf^1621^Sxl^f1^/++;eif4e^568^/+*lane 2) and *snf^1621^Sxl^f1^/++;eif4e* ***^587/11^*** */+* (lane 3) females probed with antibodies to U2AF50 and Sxl. B, C, D) RT-PCR was performed on adult females to analyze the products of the *Sxl* (B, C) or *msl-2* (D) gene. Presence or absence of an *eif4e* mutation is indicated above the relevant lanes. Results were visualized with by Southern blot (B,C) or ethidium bromide (D,E & F). Wild-type males (WT-M) produce male (ex3 included) but no female (ex3 excluded) *Sxl* mRNAs when assayed with primers that amplify only the male transcript (E.F) or primers that amplify both the male and female mRNAs (B,D). Wild-type females (WT-F) express no male *Sxl* mRNA. Females heterozygous for mutations in *snf* and *Sxl* (*snf Sxl*) express a small amount of male *Sxl* mRNA (B). Females triply heterozygous for mutations in *snf*, *Sxl* and *eif4e* express significantly more male *Sxl* mRNA. Similarly, addition of an *eif4e* mutation increases the amount of male (intron removed) *msl-2* mRNA (C). Though all of the *msl-2* mRNA in these triply heterozygous females appears to be spliced in the male pattern, there is not an obvious effect on their viability. This is not altogether surprising as females can tolerate an *hsp83* transgene that expresses an *msl-2* mRNA lacking not only the 5′ but also the 3′ Sxl binding sites (26). Panels D, E and F show that male spliced *Sxl* and *Sxl* reporter mRNAs are present in female heterozygous for two different hypomorphic *eif4e* alleles while they absent in wild type females (WT-F). In D, primers in exon2 and exon4 that amplify both male and female spliced mRNAs were used for the PCR. In E we used primers in exon3 and exon4 that amplify male spliced *Sxl* mRNAs. For the splicing reporter in F, we did two PCR reactions using nested primers in LacZ. *eif4e* alleles: *4e-68: eif4e^568^*; *4e-87: eif4e^587/11^*.

In previous studies on *snf* we found that though there is substantial female lethality when *snf^1621^/+* mothers are mated to *Sxl* ^−^ fathers, the surviving *snf^1621^/Sxl* ^−^ *trans*-heterozygous females are morphologically normal, fertile, and express wild type levels of Sxl protein**.** When we examined the splicing of the *Sxl-Pm* mRNAs in these surviving females using RT-PCR primer sets that give products spanning the regulated exon2-exon3-exon4 cassette, we found that unlike wild type females (which give only female spliced transcripts: exons 2–4) we could often detect a very low level of male spliced transcripts (exons 2–3–4) in these *snf^1621^/Sxl* ^−^ *trans*-heterozygous adult females (not shown: see *snf^1621^ Sxl^f1^/++* in Figure 4B). We reasoned that the *snf^1621^Sxl^f1^/++* heterozygous mutant combination might provide a suitable sensitized background to test whether *eif4e* activity is required for *Sxl* dependent pre-mRNA splicing.

Before assaying the splicing of *Sxl-Pm* transcripts in adult females triply heterozygous for *snf^1621^*, *Sxl^f1^,* and *eif4e*, we examined Sxl protein expression in these females. We anticipated that as long as the level of female spliced *Sxl* mRNAs remained well above some threshold critical for maintaining the positive autoregulatory feedback loop, the homeostasis mechanism provided by *Sxl* negative autoregulation of *Sxl* mRNA translation would ensure that Sxl levels would be maintained close to that in wild type. With the possible caveat that there may be tissue specific variations in Sxl levels that can't be detected by this assay, Figure 4A shows that this expectation is correct. We find that the level of Sxl protein in the triple mutant combinations with two different *eif4e* alleles is equivalent to that seen in control *snf^1621^ Sxl^f1^/++* (ane +) adult females.

We next asked if a reduction in *eif4e* activity in the sensitized *snf^1621^Sxl^fl^/++* background had any effect on the splicing of *Sxl-Pm* pre-mRNAs. For this purpose, we used a primer set that simultaneously amplifies both the male (exon 2–3–4) and female (exon 2–4) spliced *Sxl* mRNAs. This allows us to directly compare the relative ratio of female to male spliced mRNAs in each genetic background. Figure 4B shows that the very modest defects in the female specific splicing of *Sxl-Pm* pre-mRNAs evident in *snf^1621^Sxl^f1^/++* females are clearly exacerbated when *eif4e* activity is reduced. For both *eif4e* alleles there is a marked increase in the amount of male-spliced *Sxl-Pm* mRNAs compared to the *snf^1621^Sxl^f1^/++* control.

### Effects of *eif4e* mutations on msl-2 pre-mRNA splicing in a sensitized background

We used this same sensitized background to examine the effects of reducing *eif4e* activity on the splicing of the intron in the 5′ UTR of *msl-2* mRNAs. As illustrated in Figure 4C, Sxl blocks the splicing of the 5′ UTR intron so that it is retained in most *msl-2* mRNAs in females, while this intron is spliced out efficiently in males. In control *snf^1621^Sxl^f1^/++* females the female-specific splicing of the *msl-2* mRNA is partially compromised and, we observe a nearly equal mixture of female and male spliced transcripts. As observed for *Sxl-Pm* splicing, reducing *eif4e* activity in this sensitized background further disrupts the female specific splicing of *msl-2* mRNAs. In addition to demonstrating a role for *eif4e* in the splicing of a second *Sxl* target pre-mRNA, these findings provide additional evidence that the male lethal effects of the *hsp83:Sx-NΔ* transgene are suppressed because *eif4e* mutations perturb the female specific splicing of *msl-2* mRNAs.

### Male spliced Sxl mRNAs are also observed in *eif4e/+* females

The results in the previous sections demonstrate that the modest defects in *Sxl* and *msl-2* pre-mRNA splicing evident in a sensitized *snf^1621^ Sxl^f1^/++* background are significantly enhanced by reducing *eif4e* activity. We wondered whether splicing defects are also observed in *eif4e/+* females that are wild type for both *snf* and *Sxl*. To test this possibility, we examined the splicing of transcripts from the endogenous *Sxl* gene and the *Sxl* splicing reporter in females heterozygous for two different *eif4e* alleles. When we used primers that allow us to visualize simultaneously both the male and female spliced *Sxl* mRNAs from either the endogenous gene (Figure 4D) or from the splicing reporter (not shown), only female spliced *Sxl* mRNAs were observed in wild type females. In contrast, a very small amount of *Sxl* mRNA spliced in the male pattern could be detected from the endogenous gene (Figure 4D) and also from the splicing reporter (not shown) in females heterozygous for *eif4e^568^* or for *eif4e^587/11^*. To confirm that male spliced *Sxl* mRNAs from the endogenous gene are present in these *eif4e/+* females we used RT primers from exon 5 and then PCR amplified using a primer from the male exon and a primer from exon4. Figure 4E shows that male spliced *Sxl* mRNAs from the endogenous gene are readily evident in both *eif4e^568^/+* and *eif4e^587/1^/+* females, but not in wild type. Figure 4F shows that male spliced *Sxl* mRNAs from the reporter are also present in these *eif4e* heterozygous females, while there is little male spliced reporter mRNAs in control wild type females.

### Mutations in *eif4E* do not affect the alternative splicing of *dsx* mRNA

To determine whether the effects of *eif4e* on sex-specific splicing are general or only restricted to *Sxl* dependent alternative splicing we examined the splicing of *doublesex* (*dsx*) mRNAs. The *dsx* gene is downstream of *Sxl* and like *Sxl* its transcripts are sex-specifically spliced. However, female-specific splicing of *dsx* mRNA is dependent upon *tra* and *tra-2*, not *Sxl* (reviewed in [14]–[16]). We used primer sets that would RT-PCR amplify either female or male spliced *dsx* mRNAs isolated from either wild type or *eif4e/+* females. As expected, wild type females produce only female, not male products (Figure S2). Significantly, females heterozygous for *eif4e* also produce only female *dsx* mRNAs.

### Could eIF4E play a direct role in *Sxl*-dependent alternative splicing?

The results described in the previous sections show that *eif4e* is required for *Sxl* splicing. Since *eif4e* is known to function in translation initiation, it might be needed for the synthesis of some limiting *Sxl* co-factor. In this scenario, the amount of this splicing co-factor would drop below some critical threshold when *eif4e* activity is reduced, and this would impair the ability of *Sxl* to regulate splicing. Alternatively, *eif4e* itself could be the *Sxl* splicing co-factor. This latter model makes several predictions that we have tested below.

#### *eif4e* mutations enhance the female specific lethality of dominant negative *snf^1621^* and *fl(2)d^1^*

If *eif4e* functions in *Sxl* dependent alternative splicing, we might expect genetic interactions between *eif4e* and genes like *snf* that are required for female specific splicing of *Sxl* pre-mRNAs. To test this possibility females *trans*-heterozygous for different *eif4e* alleles and *snf^1621^* were mated to *Sxl^f1^* or *Sxl^7BO^* males. When combined with the *Sxl^f1^*, the weaker *eif4e^568^* allele reduces the viability of female progeny of *snf^1621^/+* mothers two-fold, while the stronger *eif4e^587/11^*allele reduces female viability ten-fold (see Table 1). An equivalent synergistic maternal effect female lethality is observed in progeny of *snf^1621^/+; eif4e^587/11^/+* mothers mated to fathers carrying the deletion allele *Sxl^7B0^*. We also observed weak, female lethal interactions when *eif4e* was combined with a mutation in another *Sxl* splicing co-factor *fl(2)d*, which encodes the fly Wilm's Tumor 1 Associated Protein (WTAP) [51], [59]–[61].

#### eIF4E is localized in the nucleus of somatic cells but not germ cells

**Figure 5:**
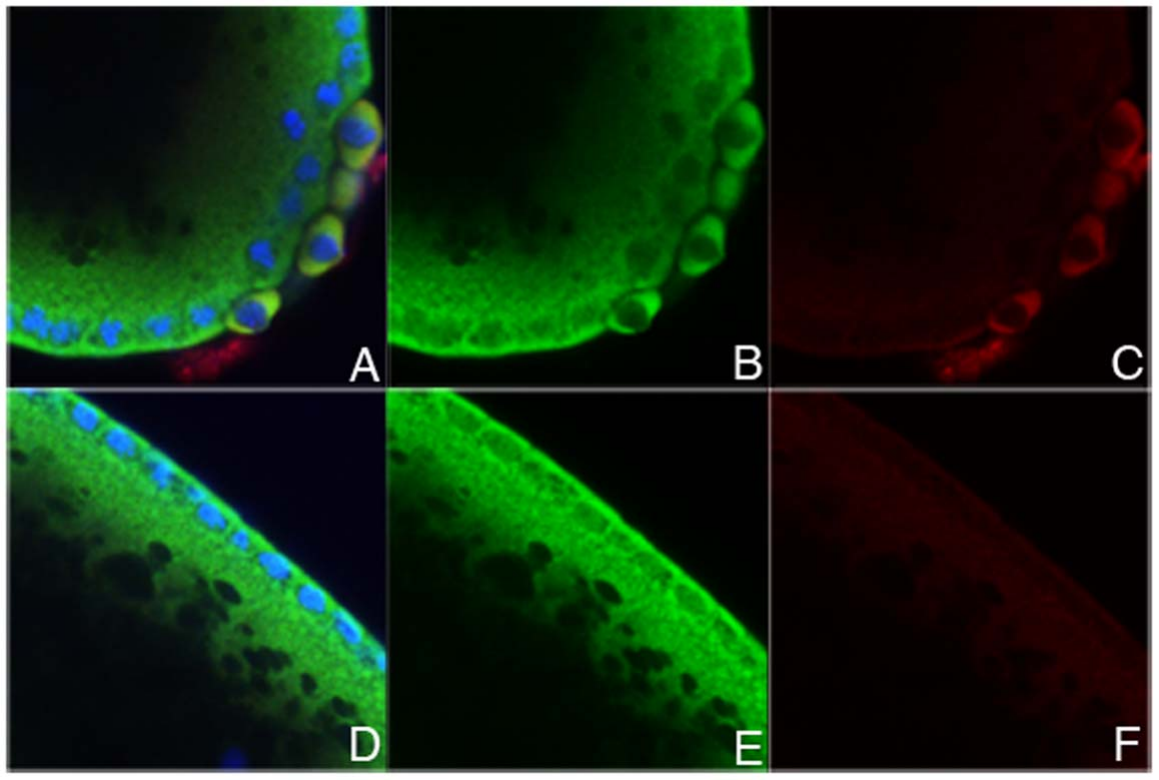
Some eIF4E protein is located in the nucleus. Wild type pre-cellular and cellular blastoderm stage embryos were stained with eIF4E (green) or Vasa (red) antibodies and hoechst (blue) to label the DNA. The embryo shown here is a late pre-cellular blastoderm embryo. A, D: All three channels. B,E: eIf4e only. C, F: Vasa only. Note the high levels of cyoplasmic eIF4E in the soma and in the Vasa positive germline pole cells. eIF4E can also be readily detected in the somatic nuclei, though the levels are less than in the somatic cytoplasm (see panel E). By contrast, there is only little eIF4E in the pole cell nuclei (Vasa plus cells at posterior in panel B).

A splicing function requires that some eIF4E protein be present in the nucleus. To test this we probed late pre-cellular and cellular blastoderm embryos with antibodies against eIF4E and the germline marker Vasa. This is the stage in development when the first *Sxl-Pm* transcripts are expressed and the positive autoregulatory feedback loop must be set in motion in females [62]. There are also marked differences in RNA polymerase activity between the soma and germline. In the soma, transcription is substantially upregulated following the midblastula transition. By contrast, newly formed germ cells are transcriptionally quiescent and genes specifying somatic development, including *Sxl*, are off. Figure 5 shows that as expected for a translation factor, most of the eIF4E in soma is localized in the cytoplasm. However, as has been reported for *Drosophila* S2 tissue culture cells [8], there is a small but readily detectable amount of eIF4E in somatic nuclei. Interestingly, the transcriptionally quiescent germ cells differ from the somatic cells in that eIF4E is exclusively cytoplasmic and is not observed in their nuclei.

#### eIF4E is bound to *Sxl* pre-mRNAs

**Figure 6:**
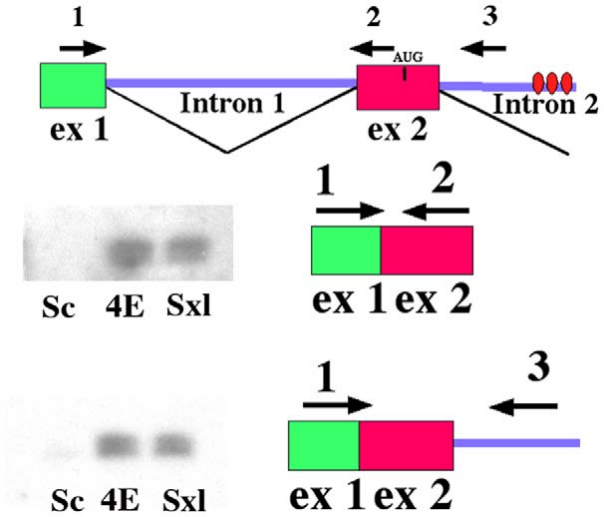
eIF4E co-immunoprecipitates with *Sxl* pre-mRNAs. Nuclear extracts were incubated with antibodies to Scute (Sc), Sxl or eIF4E (4E). RNA was isolated from the immunoprecipitates and used for RT-PCR reactions. Top: Diagram of the 5′ region of the *Sxl-Pm* transcription unit showing the exon-intron structure and the position of primers used for PCR. Bottom: Southern blots of RT-PCR products that are amplified from the immunoprecipitates using the indicated primers. Next to the blots is a diagram of the amplification product. Antibodies to eIF4E and Sxl immunoprecipitate both spliced and partially spliced *Sxl-Pm* mRNAs. Antibodies to Sc do not immunoprecipitate any *Sxl-Pm* mRNAs.

To function in *Sxl* dependent alternative splicing, eIF4E has to be bound to incompletely spliced *Sxl* transcripts. We first tested for the binding of Sxl and eIF4E to nuclear *Sxl* RNAs that had undergone the first splice of exon 1 to exon 2. As shown in the top panel of Figure 6, exon 1–2 spliced *Sxl* RNAs are found associated with both Sxl and eIF4E in nuclear extracts. Since splicing of the regulated sex-specific exons in the *Sxl-Pm* pre-mRNA is known to occur more slowly than the splicing of the non-regulated exons in the transcript [63], we next assayed the immunoprecipitates for *Sxl-Pm* RNAs in which exon1 has been spliced to exon2, but the Sxl regulated splice between exon2 and either exon3 or exon4 has not yet occurred (see 2^nd^ panel in Figure 6). Consistent with previous studies which have shown that Sxl binds to partially spliced RNAs [43], exon1-exon2-intron2 *Sxl-Pm* RNAs are found in Sxl immunoprecipitates. Consistent with a function in the sex-specific splicing of *Sxl-Pm* pre-mRNAs, exon1-exon2-intron2 *Sxl-Pm* RNAs are also found in eIF4E immunoprecipitates, but not in control Scute immunoprecipitates. To exclude the possibility that Sxl and eIF4E associate non-specifically with any pre-mRNA in nuclear extracts, we assayed for the presence of incompletely processed *tango* transcripts; however, unspliced *tango* RNAs were not detected in either Sxl or eIF4E immunoprecipitates (data not shown). Since we were able to detect *tango* pre-mRNAs in U2UF50 immunoprecipitates, it would appear that eIF4E does not bind to all pre-mRNAs.

#### eIF4E is associated with Sxl and Snf in nuclear extracts

**Figure 7:**
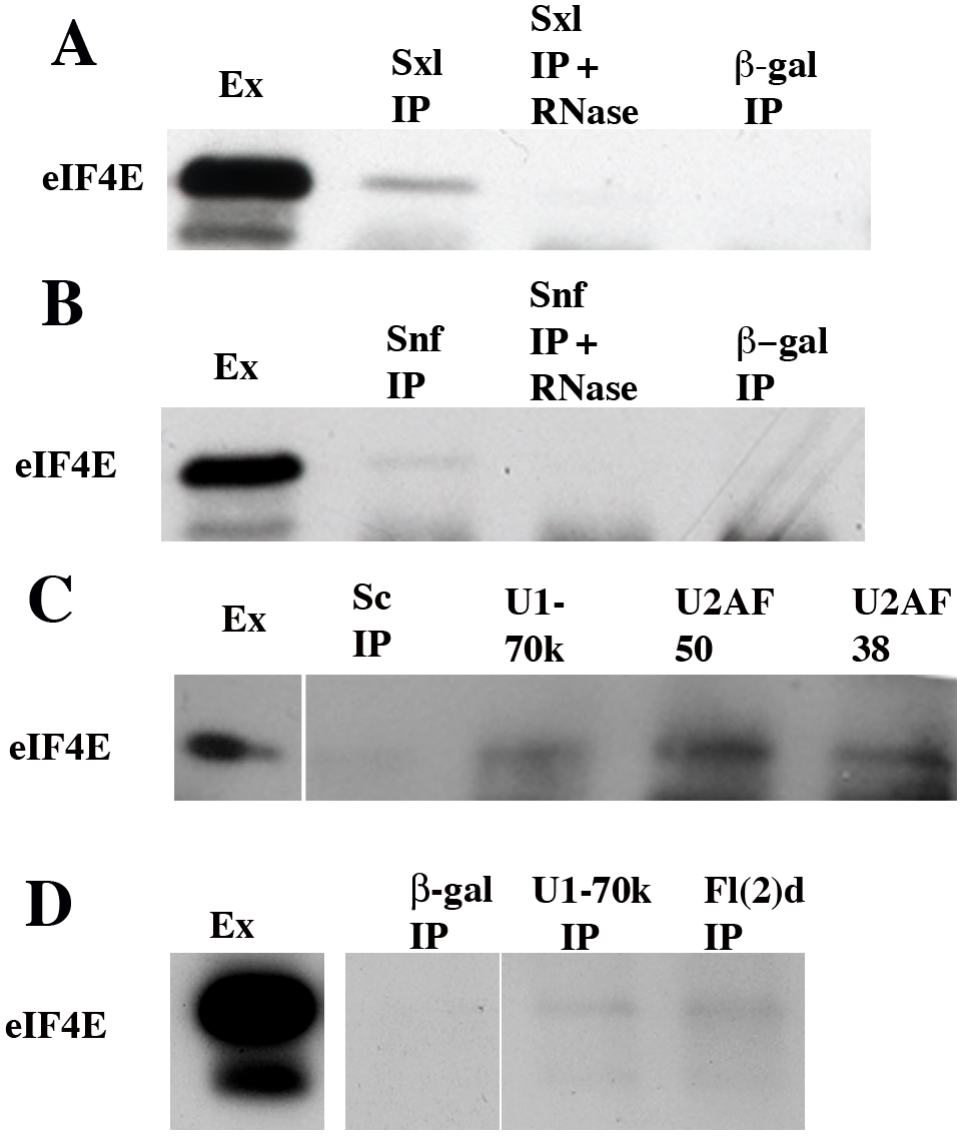
eIF4E co-immunoprecipitates with several splicing factors. Western blots of immunoprecipitates isolated using antibodies to splicing factors (Sxl, Snf, U1-70K, U2AF50, U2AF38, and Fl(2)d) or negative controls (β-Galactosidase or Scute (Sc)) were probed with antibodies against eIF4E. Nuclear extract (lane 1 all blots) contains substantial amounts of eIF4E. Two isoforms are usually observed in nuclear extracts; however, the lower isoform is often obscured by the immunoglobulin light chain in the IPs. A, B) eIF4E is present in Sxl and Snf immunoprecipitates (2^nd^ lane from left in panels A and B as indicated), but is released from the Sxl and Snf complexes by pre-treatment with RNase (3^rd^ lane from left as indicated). eIF4E is not immunoprecipitated by antibodies to β-galactosidase (lane 4 from left). Note that though Snf and Sxl interact directly with each other *in vitro* and *in vivo*, Sxl protein is typically not detected in Snf immunoprecipitates of total nuclear extracts (37) whereas Snf is readily detected in Sxl IPs. The reason for this discrepancy is that only a small amount of the Snf protein is associated with Sxl. As we are able to recover only a fraction of the total Snf in the IPs, there is probably too little Sxl to be detected. On the other hand, Sxl can be readily detected in the Snf IPs when Sxl:Snf complexes are first partially purified away from bulk Snf protein on sucrose gradients and then immunoprecipitated. (C) eIF4E is not present in Scute (Sc) immunoprecipitates (2^nd^ lane), but is present in the U1-70K, U2AF50 and U2AF38 immunoprecipitates (lanes 3–5 from left as indicated). (D) eIF4E is not present in β-galactosidase immunoprecipitates (2^nd^ lane), but is present in the U1-70k (3^rd^ lane from left) and Fl(2)d (4^th^ lane from left) immunoprecipitates. Band visible at very bottom of the IP lanes in panels A, B and also C is the immunoglobulin light chain.

If eIF4E participates in *Sxl* dependent splicing regulation, it should be associated not only with Sxl but also with the U1/U2 snRNP protein that has been implicated *Sxl* splicing regulation. As can be seen in Figure 7A, eIF4E is present in Sxl, but not control immunoprecipitates of nuclear extracts. Similarly, a small but readily detectable amount of eIF4E is found in the Snf immunoprecipitates (Figure 7B). Though recombinant Sxl and Snf are able to interact directly with each other *in vitro*, the complexes between these two proteins in nuclear extracts are disrupted by RNase digestion [43]. Figure 7A and 7B show that like nuclear Sxl:Snf complexes, both the eIF4E:Sxl and eIF4E:Snf complexes are also RNase sensitive.

#### eIF4E is associated with splicing factors that function in the assembly of the spliceosome complexes E and A

We [43], [61] and Nagengast *et al* [45] have presented genetic and biochemical evidence that *Sxl* autoregulation depends upon interactions between Sxl and components of the splicing machinery that are involved in the initial assembly of the U1 snRNP on the 5′ splice sites of the *Sxl-Pre* mRNAs and the U2 snRNP on the 3′ splice sites. If eIF4E plays a role in *Sxl* autoregulation, it should also be present in RNP complexes that contain factors that function at these early steps in the splicing reaction. Both U1-70K, which is a component of the U1 snRNP and the U2AF proteins, U2AF38 and U2AF50 play important roles in *Sxl* autoregulation and are found associated with Sxl protein in nuclear extracts [45]. U1-70K/U1 snRNP and the U2AF heterodimer function in one of the first steps in the splicing reaction, the formation of the E complex. This complex is formed by the binding of the U1-70K/U1 snRNP to the 5′ splice site and the interaction of the U2AF heterodimer with the polypyrimidine tract at the 3′ splice junction. U2AF at the 3′ splice site then recruits the U2 snRNP which becomes loosely associated with the pre-mRNA [64]–[69]. The E spliceosome complex then undergoes an ATP dependent rearrangement that stabilizes the pairing interactions between the U2 snRNP and the pre-mRNA to form the A complex [70]–[73]. U1-70K/U1 snRNP and U2AF remain associated with the splicing complex when the three other snRNPs, U4/U6 and U5, are recruited to give spliceosomal complex B [74]; however, when the B complex rearranges during formation of the activated complex B* both U1-70K and U2AF dissociate from the splicesome along with the U1 and U4 snRNPs [75]–[77]. To determine if eIF4E is associated with these early acting splicing factors, we immunoprecipitated nuclear extracts with antibodies against U1-70K and the two U2AF subunits dU2AF38 and dU2AF50. Figure 7C and 7D show that eIF4E is in complexes in the nucleus with the U1-70K protein and with both of the U2AF subunits.

Another factor required for Sxl regulated splicing is the fly WTAP protein Fl(2)d [59]–[60], [77]. The interaction of Fl(2)d with the spliceosomal apparatus more closely parallels that seen for Sxl than U1-70K, U2AF or Snf. Like Sxl, Fl(2)d is found associated with splicing factors that are present during the formation of the spliceosomal E and A complexes which define the 5′ and 3′ exon-intron junctions and position the U2 snRNP, but appears to disassociate from the spliceosome before the tripartite snRNPs, U4/U6 and U5, are recruited to the pre-mRNA to form pre-catalytic complex B. The available evidence indicates that Sxl:Fl(2)d interactions may facilitate the incorporation of Sxl into pre-mRNA spliceosome complexes and perhaps mediate its interactions with Snf [61]. If eIF4E is important for *Sxl* dependent alternative splicing, we would expect to find it associated not only with Sxl but also with Fl(2)d in nuclear extracts. Figure 7D shows that this prediction is correct: eIF4E can be co-immunoprecipitated with Fl(2)d.

## Discussion

The RNA binding protein Sxl orchestrates sexual development by controlling gene expression post-transcriptionally at the level of splicing and translation. To exert its different regulatory functions Sxl must collaborate with sex-non-specific components of the general splicing and translational machinery. In the studies reported here we present evidence that one of the splicing co-factors is the cap binding protein eIF4E. We initially identified *eif4e* in a screen for mutations that dominantly suppress the male lethal effects induced by ectopic expression of a mutant Sxl protein, Sx-N, which lacks part of the N-terminal domain. The Sx-N protein is substantially compromised in its splicing activity, but appears to have closer to wild type function in blocking the translation of the *Sxl* targets *msl-2* and *Sxl-Pm*. As the male lethal effects of Sx-N (in an *Sxl^-^* background) are due to its inhibition of Msl-2 expression [39] we anticipated that general translation factors needed to help *Sxl* repress *msl-2* mRNA would be recovered as suppressors in our screen. Indeed, one of the suppressors identified was *eif4e*. However, consistent with *in vitro* experiments, which have shown that Sxl dependent repression of *msl-2* mRNA translation is cap independent [34], we found that *eif4e* does not function in Sxl mediated translational repression of at least one target mRNA *in vivo*. Instead, our results indicate that *eif4e* is needed for *Sxl* dependent alternative splicing and argue that it is this splicing activity that accounts for the suppression of male lethality by *eif4e* mutations. In wild type females, Sxl protein blocks the splicing of a small intron in the 5′ UTR of the *msl-2* pre-mRNA. This is an important step in *msl-2* regulation because the intron contains two Sxl binding sites that are needed by Sxl to efficiently repress translation of the processed *msl-2* mRNA. When this intron is removed repression of *msl-2* translation by Sxl is incomplete [25]–[28] and this would enable *eif4e/+* males to escape the lethal effects of the *Sx-N* transgene.

Several lines of evidence support the conclusion that *eif4e* is required for *Sxl* dependent alternative splicing. One comes from our analysis of the dominant maternal effect female lethal interactions between *eif4e* and *Sxl*. The initial activation of the *Sxl* positive autoregulatory feedback loop in early embryos can be compromised by a reduction in the activity of splicing factors like Snf, Fl(2)d, and U1-70K, and mutations in genes encoding these proteins often show dose sensitive maternal effect, female lethal interactions with *Sxl*. Like these splicing factors, maternal effect female lethal interactions with *Sxl* are observed for several *eif4e* alleles. Moreover, these female lethal interactions can be exacerbated when the mothers are *trans*-heterozygous for mutations in *eif4e* and the splicing factors *snf* or *fl(2)d*. Genetic and molecular experiments indicate that female lethality is due to a failure in the female specific splicing of *Sxl-Pm* mRNAs. First, female lethality can be rescued by gain-of-function *Sxl* mutations that are constitutively spliced in the female mode. Second, transcripts expressed from a *Sxl-Pm* splicing reporter in the female *Sxl* ^−^ */+* progeny of *eif4e/+* mothers are inappropriately spliced in a male pattern at the time when the *Sxl* positive autoregulatory loop is being activated by the Sxl-Pe proteins. While splicing defects are evident in these embryos at the blastoderm/early gastrula stage, obvious abnormalities in expression of Sxl protein are not observed until several hours later in development.

Though this difference in timing would favor the idea that *eif4e* is required for splicing of *Sxl-Pm* transcripts rather than for the export or translation of the processed *Sxl-Pm* mRNAs, we can not exclude the possibility that there are subtle defects in the expression of Sxl protein at the blastoderm/early gastrula stage that are sufficient to disrupt splicing regulation during the critical activation phase yet aren't detectable in our antibody staining experiments. However, evidence from two different experimental paradigms using adult females indicates that this is likely not the case. In the first, we found that reducing *eif4e* activity in a sensitized *snf^1621^ Sxl^f1^/++* background can compromise *Sxl* dependent alternative splicing even though there is no apparent reduction in Sxl protein accumulation. In this experiment we took advantage of the fact that once the positive autoregulatory feedback loop is fully activated a homeostasis mechanism (in which *Sxl* negatively regulates the translation of *Sxl-Pm* mRNAs) ensures that Sxl protein is maintained at the same level even if there are fluctuations in the amount of female spliced mRNA. While only a small amount of male spliced *Sxl-Pm* mRNAs can be detected in *snf^1621^ Sxl^f1^/++* females, the level increases substantially when *eif4e* activity is reduced. Since these synergistic effects occur even though Sxl levels in the triply heterozygous mutant females are the same as in the control *snf^1621^ Sxl^f1^/++* females, we conclude that the disruption in *Sxl* dependent alternative splicing of *Sxl-Pm* transcripts in this context (and presumably also in early embryos) can not be due to a requirement for *eif4e* in either the export of *Sxl* mRNAs or in their translation. Instead, *eif4e* activity must be needed specifically for *Sxl* dependent alternative splicing of *Sxl-Pm* pre-mRNAs. Consistent with a more general role in *Sxl* dependent alternative splicing, there is a substantial increase in *msl-2* mRNAs lacking the first intron when *eif4e* activity is reduced in *snf^1621^ Sxl^f1^/++* females. In the second experiment we examined the splicing of pre-mRNAs from the endogenous *Sxl* gene and from a *Sxl* splicing reporter in females heterozygous for two hypomorphic *eif4e* alleles. Male spliced mRNAs from the endogenous gene and from the splicing reporter are detected the *eif4e/+* females, but not in wild type females. Moreover, the effects on sex-specific alternative splicing seem to be specific for transcripts regulated by *Sxl* as we didn't observe any male spliced *dsx* mRNAs in *eif4e/+* females.

Two models could potentially explain why *eif4e* is needed for *Sxl* dependent alternative splicing. In the first, *eif4e* would be required for the translation of some critical and limiting splicing co-factor. When *eif4e* activity is reduced, insufficient quantities of this splicing factor would be produced and this, in turn, would compromise the fidelity of *Sxl* dependent alternative splicing. In the second, the critical splicing co-factor would be *eif4e* itself. It is not possible to conclusively test whether there is a dose sensitive requirement for *eif4e* in the synthesis of a limiting splicing co-factor. Besides the fact that the reduction in the level of this co-factor in flies heterozygous for hypomorphic *eif4e* alleles is likely to be rather small, only a subset of the *Sxl* co-factors have as yet been identified (unpublished data). For these reasons, the first model must remain a viable, but in our view, unlikely possibility. As for the second model, the involvement of a translation factor like *eif4e* in alternative splicing is unexpected if not unprecedented. For this to be a viable model, a direct role for *eif4e* must be consistent with what is known about the dynamics of *Sxl* pre-mRNA splicing and the functioning of the Sxl protein. The evidence that the second model is plausible is detailed below.

Critical to the second model is both the nuclear localization of eIF4E and an association with incompletely spliced *Sxl* pre-mRNAs. Nuclear eIF4E has been observed in other systems, and we have confirmed this for *Drosophila* embryos. We also found that eIF4E is bound to *Sxl* transcripts in which the regulated exon2-exon3-exon4 cassette has not yet been spliced. In contrast, it is not associated with incompletely processed transcripts from the *tango* gene, which are constitutively spliced. With the caveat that we have only one negative control, it is not surprising that *Sxl* transcripts might be unusual in this respect. There is growing body of evidence that splicing of constitutively spliced introns is co-transcriptional [78]–[83]. However, recent *in vivo* imaging experiments have shown that the splicing of the regulated *Sxl* exon2-exon3-exon4 cassette is delayed until after the *Sxl* transcript is released from the gene locus in female, but not in male cells [84]. These *in vivo* imaging studies also show that, like bulk pre-mRNAs, the 1^st^ *Sxl* intron is spliced co-transcriptionally in both sexes. Consistent with a delay in the splicing of the regulated cassette, we've previously reported that polyadenylated *Sxl* RNAs containing introns 2 and 3 can be readily detected by RNase protection, whereas other *Sxl* intron sequences are not observed [19]. The delay in the splicing of the regulated *Sxl* cassette until after transcription is complete and the RNA polyadenylated could provide a window for exchanging eIF4E for the nuclear cap binding protein.

To function as an *Sxl* co-factor, eIF4E would have to be associated with the pre-mRNA-spliceosomal complex before or at the time of the Sxl dependent regulatory step. There is still a controversy as to exactly which step in the splicing pathway Sxl exerts its regulatory effects on *Sxl-Pm* pre-mRNAs and two very different scenarios have been suggested. The first is based on an *in vitro* analysis of *Sxl-Pm* splicing using a small hybrid substrate consisting of an Adenovirus 5′ exon-intron fused to a short *Sxl-Pm* sequence spanning the male exon 3′ splice site [85]. These *in vitro* studies suggest that Sxl acts very late in the splicing pathway after the 1^st^ catalytic step, which is the formation of the lariat intermediate in the intron between exon 2 and the male exon. According to these experiments Sxl blocks the 2^nd^ catalytic step, the joining of the free exon 2 5′ splice site (or Adeno 5′ splice site) to the male exon 3′ splice site (see Figure 1A). It is postulated that this forces the splicing machinery to skip the male exon altogether and instead join the free 5′ splice site of exon 2 to the downstream 3′ splice site of exon 4. Since we have shown that eIF4E binds to *Sxl-Pm* pre-mRNAs that have not yet undergone the 1^st^ catalytic step (Figure 6), it would be in place to influence the splicing reaction if this scenario were correct.

The second scenario is more demanding in that it proposes that *Sxl* acts during the initial assembly of the spliceosome. Evidence for Sxl regulation early in the pathway comes from the finding that Sxl and the Sxl co-factor Fl(2)d show physical and genetic interactions with spliceosomal proteins like U1-70K, Snf, U2AF38 and U2AF50 that are present in the early E and A complexes and are important for selecting the 5′ and 3′ splice sites [45], [61], [64]–[71]. In addition to these proteins, Sxl can also be specifically cross-linked in nuclear extracts to the U1 and U2 snRNAs [43]. Formation of the E complex depends upon interactions of the U1 snRNP with the 5′ splice site, and this is thought to be one of the first steps in splicing. The other end of the intron is recognized by U2AF, which recruits the U2 snRNP to the 3′ splice site. After the base pairing of the U2 snRNP with the branch-point to generate the A complex the next step is the addition of the U4/U5/U6 snRNPs to form the B complex. However, Sxl and Fl(2)d are not found associated with components of the splicing apparatus like U5-40K, U5-116K or SKIP that are specific for complexes B and B*, or the catalytic C complex [70]–[71], [74]–[75], [86]–[88]. Nor can Sxl be cross-linked to the U4, U5 or U6 snRNAs [43]. If Sxl and Fl(2)d dissociated from the spliceosome before U4/U5/U6 are incorporated into the B complex, then they must influence splice site selection during the formation/functioning of the E and/or A complex. (Since the transition from the E to the A complex has been shown to coincide with an irreversible commitment to a specific 5′—3′ splice site pairing, Sxl would likely exerts its effects in the E complex when splice site pairing interactions are known to still be dynamic [89].) If this is scenario is correct, eIF4E would have to be associated with factors present in the earlier complexes in order to be able to promote *Sxl* regulation. This is the case. Thus, eIF4E is found in complexes containing the U1 snRNP protein U1-70K, the U1/U2 snRNP protein Snf, and the two U2AF proteins, U2AF38 and U2AF50. With the exception of the Snf protein bound to the U2 snRNP, all of these eIF4 associated factors are present in the early E or A complexes, but are displaced from the spliceosome together with the U1 and U4 snRNPs when the B complex is rearranged to form the activated B* complex. This would imply that eIF4E is already in place either before or at the time of B complex assembly. Arguing that eIF4E associates with these E/A components prior to the assembly of the B complex is the finding that eIF4E is also in complexes with both Sxl and Fl(2)d. Thus, even in this more demanding scenario for *Sxl* dependent splicing, eIF4E would be present at a time when it could directly impact the regulatory activities of Sxl and its co-factor Fl(2)d.

Taken together these observations would be consistent with a Sxl co-factor model. While further studies will be required to explain how eIF4E helps promote female specific processing, an intriguing possibility is suggested by the fact that hastening the nuclear export of *msl-2* in females would favor the female splice (which is no splicing at all). Hence, one idea is that eIF4E binding to the pre-mRNA provides a mechanism for preventing the *Sxl* regulated splice sites from re-entering the splicing pathway, perhaps by constituting a “signal” that blocks the assembly of new E/A complexes. A similar post-transcriptional mechanism could apply to female-specific splicing of the regulated *Sxl* exon2-exon3-exon4 cassette. The binding of eIF4E (and PABP) to incompletely processed *Sxl* transcripts after transcription has terminated in females would prevent the re-assembly of E/A complexes on the two male exon splice sites, and thus promote the formation of an A complex linking splicing factors assembled on the 5′ splice sites of exons 2 and on the 3′ splice site of exon 4.

## Materials and Methods

### Fly culture

Flies were raised at room temperature on a standard Drosophila media. Crosses were performed at 29°C unless otherwise indicated with 3–7 females and 2–4 males per vial. Crosses were transferred to new vials every 2–3 days. Similar crosses were performed at 25°C, but the effects were significantly weaker.

### Stocks

Unless otherwise noted stocks are referenced by Lindsley & Zimm [89]. *w; eif4e^SO587/11^/TM3Sb (eif4e^587^*, FBal0129763*), w;eif4e^EP568^/TM3Sb (eif4e^568^*, FBal0122994*), w;eif4e^SO715^/TM3Sb(eif4e^715^*, FBal0175695*), y^1^w^67c23^, w cm Sxl^f1^ ct/Bincinscy, y w* (FBal0016680), *Sxl^7BO^/Bincinscy* (FBal0016694), *y pn Sxl^M1^/Bincinscy* (FBal0016703), *y pn Sxl^M4^/Bincinscy* (FBal0016710), *y pn Sxl^M6^/Bincinscy* (FBal0103944), *cm Sxl^f9^/Bincinscy* (FBal0016686), *y w sn^f1621^ ct/Bincinscy, y w snf^1621^ Sxl^f1^ ct/Bincinscy.*

### Screen for suppressors of *hsp83:Sx-NΔ* transgene

To identify suppressors of the dominant male lethality conferred by Sx-N, we crossed *w Sxl^7B0^/Bin; hsp83:Sx-NΔ* transgene mothers to Deficiency/Balancer fathers and scored for viable, non-Balancer males containing the transgene. The 67A8-A9 region was one of the chromosomal intervals that was found to contain a suppressor. The *eif4e* gene mapped to this region and was a strong candidate gene for the dominant suppressor. Four independent *eif4e* alleles suppressed the male lethal effects of *hsp83:Sx-NΔ* transgene as indicated in the text. All crosses for both screens were conducted in vials with five females and three males of the appropriate genotype. Matings were allowed to occur for three days at 25°C, at which time the parents were transferred to new vials to ensure that larvae were not crowded.

### Immunohistochemical staining

Embryos were collected on apple juice plates sprinkled with yeast at 29°C. They were dechorionated in bleach and fixed in 4% paraformaldehyde:heptane for 20–25 minutes. The fix was removed and embryos devitilinized and stored in methanol at −20°C. To stain, embryos were stepped into PBS, incubated for 1 hour in PAT (PBS with 1% BSA, 1%Triton-X100) and blocked for 30 minutes in PBT (PBS with 5% BSA). Embryos were incubated overnight at 4°C with primary antibody at the appropriate concentration in PBT. The next day the embryos were washed with PBS-T (PBS with 1% Triton-X100) then, incubated for 2 hours at room temperature with secondary antibody at the appropriate concentration in PBT. Embryos were washed with PBS-T, then with PBS. For embryos with fluorescently tagged secondary antibodies, the embryos were incubated for 5 minutes with a 1∶1000 dilution of Hoescht, rinsed twice with PBS, then mounted in Aquamount (Polysciences, Inc.). For embryos with HRP conjugated secondary antibodies, embryos were incubated with 400 ul of 0.4 mg/ml DAB in PBS, 1 ul of 3% hydrogen peroxide and 0.6 ul of 1 M NiCl2 until the embryos appeared fully stained. To prepare for mounting embryos were stepped into 100% ethanol, then incubated overnight in methyl salicylate. The following morning, embryos were mounted in Permount (Fisher Scientific). Primary antibodies used were: anti-Sxl18 monoclonal at 1∶10, anti-snf 9G3 monoclonal at 1∶10 and anti-eIF4E polyclonal at 1∶500 (gift from Paul Lasko). Secondary antibodies used were: HRP conjugated goat anti-mouse (Jackson ImmunoResearch) at 1∶500, rhodamine conjugated goat anti-rabbit (Alexa) at 1∶500, fluorescence conjugated goat anti-mouse (Alexa) at 1∶500.

### RT-PCR analysis and Southern blotting

Embryonic RNA was prepared as described by Bell et al [90]. Adult RNA from 33 flies was prepared using GE Healthcare mini-spin columns. Reverse transcription was performed according to the procedure of Frohman et al. [91]. 1.5–3% of the cDNA was used as template. PCR cycles for embryonic cDNAs were 1X 95°C 4 minutes, 30X 95°C 1 minute, 60–65°C 45 seconds, 72°C 30 seconds, 1X 72°C 10 minutes. If re-amplification was needed, only 10 cycles were performed in the first PCR. Up to 40% of the first PCR was used as template for the second PCR. Primers and temperatures were the same for the second reaction as in the first and 10–30 cycles were performed as needed. Number of cycles needed was evaluated by examining 10 ul samples with EtBr. For adult cDNAs PCR cycles were as follows: 2X 95°C 1 minute, 70–72°C 45 seconds, 7°C 1 minute, 2-4X 95°C 1 minute, 68–70°C 45 seconds, 72°C 1 minute, 2-4X 95°C 1 minute, 66–67°C 45 seconds, 72°C 1 minute, 2-4X 95°C 1 minute, 65–66°C 45 seconds, 72°C 1 minute, 10X (first PCR) or 5-30X (second PCR) 95°C 1 minute, 65–67°C 45 seconds, 72°C 1 minute. 5 ul of the first PCR diluted 1/100 was used as template for the second PCR. For Southern blotting DNA was run on 1–1.2% agarose gels, and Southern blotted to Zeta-Probe membrane or nitrocellulose. For Sxl reactions blots were hybridized with randomly primed *Sxl* 3B1Δ cDNA [39]. For *msl-2* mRNAs the membrane was hybridized to randomly primed *msl-2* 5′UTR PCR product. Primers used are described in Figure 6 and listed in Table 2.

**Table 2:** Primers used for RT-PCR experiments.

Experiment	RT primer	PCR primers	Sequence
Splice reporterFigure 3	lacZ1		CGCATCGTAACCGTGCATCTGC
		lacZ2EX2	CGCCATTCAGGCTGCGCAACTG GTGGTTATCCCCCATATGGC
Sxl in adult femalesFigure 4B	T41-3		CGTGTCCAGCTGATCGTC
		mes17BelA	CGCTGCGAGTCCATTTCC GTGGTTATCCCCCATATGGC
Sxl in adult femalesFigure 4C	T41-3		CGTGTCCAGCTGATCGTC
		MALELPGEX8	AGAAAGAAGCAGCCACCATTATCACC ATTCCGGATGGCAGGAATGGGAC
msl-2 in adult femalesFigure 4D	948r		ATGTTTGAGCCCTCGCGAAT
		17f707r	TATGCCGCACTGXAGCTAATGCTTCTTACCGCGCAGA
IP-RT-PCRFigure 6B	T41-3		CGTGTCCAGCTGATCGTC
		Sxl1Sxl2Sxl3Sxl4	GTTGCCGAAGGAAAGTCGC TGGGAGAGCGAGCAAAAACG CCGGATTATTGTTGCCGTACATATCC GCTCTCTCACGTAGGCGC

### Immunoprecipitation

Nuclear extract was prepared by collecting embryos laid by w^1^ stock overnight (<24 hours). Embryos were washed with distilled water and 0.12 M NaCl, 0.04% Triton-X 100, then dechorionated in 100% bleach for 3 minutes. Dechorionated embryos were rinsed with NaCl, Triton, then NaCl, blotted dry and collected. Embryos were homogenized at 4°C in buffer 1(15 mM HEPES-KOH pH 7.6, 10 mM KCl, 5 mM MgCl_2_, 0.1 mMEDTA, 0.5 mM EGTA, 0.35 M sucrose, with 1 mM DTT, 1 mMNa_2_S_2_O_5_, protease inhibitors, benzamidine and 1mMPMSF), using 4 ml buffer/ml lightly packed embryos. The homogenate was filtered through three layers of Mira-cloth, then centrifuged at 2000 xg for 10 minutes at 4°C. Supernatant was removed with a pipet. The pellet was re-suspended in 4 ml buffer/ml embryos, and overlaid onto an equal volume of buffer 2 (same as buffer 1 except 0.8 M sucrose), then spun 10 minutes at 2000 xg, at 4°C. The supernatant was removed. The pellet was resuspended in TEN (10 mM Tris-HCl pH 7.8–8, 1.5 mM EDTA, 100 mM NaCl), 2 ml TEN/ml embryos, and sonicated. 20 ul–40 ul of 50% antibody linked protein AG beads, 350 ul co-immunoprecipitation buffer (20 mM Hepes, pH 7.5, 150 mM NaCl, 250 mM sucrose, 0.05% (w/v) Tergitol NP-40, 0.5% (v/v) Triton-X 100 plus 1 mM DTT, 1 mMNa_2_S_2_O_5_, protease inhibitors, benzamidine and 1mMPMSF) and 12.5 ul RNAsin were added to a 150 ul aliquot of sonicate. The mixture was rocked at 4°C overnight, then washed 5 times with co-IP buffer. The beads were boiled for 5–10 minutes with 20 ul protein sample buffer, then spun for 5–10 minutes. 5–10 ul of sample was loaded onto a 12% polyacrylimide gel. The proteins were transferred to Immobilon-P or nitrocellulose. Blots were prehybridized in PBS-5% nonfat dry milk and probed with primary antibody overnight at 4°C. Antibodies used include: mouse anti-Sxl 104 and 114, mouse anti snf 9G3 [41], rabbit anti-eIF4e antibody at 1∶1000 [92], rabbit anti-U170K (gift of Helen Salz; [45]) at 1/5000, rabbit anti-U2AF50 (gift of Don Rio; [93])at 1/5000, rabbit anti-U2F38 1/5000 (gift of Don Rio; [94]) or mouse anti-Fl(2)d9G2 [60] at 1/10, mouse anti-scute 5A10 [95]. Blots were washed three times for 10 minutes each in PBST and hybridized with horseradish peroxidase-conjugated secondary antibody (Goat anti-rabbit (1∶10,000) or Goat anti-mouse (1/1000−1/10,000) from Jackson ImmunoResearch) in PBST-5% milk for two hours at room temperature. Blots were again washed three times for 10 minutes each in PBST and visualized with an enhanced chemiluminescent agent.

### Immunoprecipitation, RT-PCR

Nuclear extract was prepared essentially as above except, after the first centrifugation, the pellet was resuspended in 1 ml buffer/ml embryos and sonicated. 20 ul of 50% antibody linked protein A beads were added to a 150 ul aliquot of sonicate. The mixture was allowed to rock 1 hour at room temperature, then washed as above. RNA was isolated using TRIreagent (Molecular Research Center, Inc.) then, treated with DNAse 1. Reverse transcription, PCR and Southern blotting conditions were as described above with primers as indicated in Figure 6 and Table 2. Southern blotting conditions were as described above using randomly primed *Sxl 3B1Δ* cDNA [39) as the probe. Antibodies used for immunoprecipitation were; anti-scute SA10, anti-Sxl 104 and 114 mixed 1∶1, and anti-eIF4E.

### Western blotting

2–5 flies of each genotype were collected and frozen at −80°C. 10 ul of 2x Laemmli sample buffer per fly was added to the flies, which were then homogenized with a hand held Dounce homogenizer. Samples were boiled for 5 minutes and spun for three minutes at 14,000 rpm. Samples were diluted as needed with 2x Laemmli sample buffer and up to 10 ul of sample were loaded onto sodium dodecyl sulfate (SDS)-12% acrylamide gels, run out and transferred to Immobilon-P or nitrocellulose. Blots were incubated for 60 minutes in PBST (PBS with 1% Triton-X100), with 10% dry milk then, incubated overnight at 4°C with primary antibody at the appropriate concentration in PBST with 10% dry milk. The next day the blots were washed with PBST for at least an hour, then incubated for 2–4 hours at room temperature with secondary antibody at the appropriate concentration in PBST with 10 mg/ml BSA. Blots were washed with PBST then, developed with ECL Plus (Amersham). Primary antibodies used were: a 1∶1 mixture of anti-SXL104 and 114 at 1/10−1/1000, anti-eIF4E 1739 at 1/1000, anti-U2AF50 at 1/50,000, and anti-dFMR J11 at 1/1000. HRP conjugated goat anti-mouse and goat anti-rabbit (Jackson ImmunoResearch) secondary anti-bodies were used at 1/2500 or 1/5000.

## Supporting Information

Figure S1 Male and female spliced *msl-2* mRNA in surviving *Sxl* ^−^ *;eif4e/+; hsp83:Sx-NΔ* males. RNA isolated from one of the 3 surviving *Sxl* ^−^ *;eif4e/+; hsp83:Sx-NΔ* males was reversed transcribed with a primer complementary to sequences in exon 3 (downstream of primer 3). We then used two different primer combinations, P1–P2 and P1–P3 to amplify female and male *msl-2* transcripts as indicated. Similar results were obtained from the two other surviving *Sxl* ^−^ *;eif4e/+; hsp83:Sx-NΔ* males.
(TIF)

Figure S2 Female specific splicing of *dsx* mRNA is unaffected by reducing *eif4e* activity. RNA from wild type males and females and females heterozgyous for either *eif4e^568^* (*4E568*) or *eif4e^587/^* ^11^ (*4E587*) were reverse transcribed with primers specific for the female spliced or male spliced 3′ UTRs. The resulting cDNAs were then PCR amplified using primers complementary to the common exon 3 and the female exon 4 (*dsx* female), or to the common exon 3 and the first male exon 5 (*dsx* male). Female specific, but not male specific amplification products are detected in wild type females and in females heterozygous for the two *eif4e* mutations. To ensure that the amplification products we are seeing are specific *dsxF* and *dsxM*, we used nested primers in the common exon for two rounds of PCR amplification.
(TIF)

Table S1 Sex-lethal staining patterns in older embryos. Unless otherwise indicated females were crossed to *Sxl^f1^* males at 29°C. Progeny were collected as embryos and stained with antibody to Sxl. Embryos at the cellular blastoderm stage or those past nuclear cycle 13 were examined and placed into one of the indicated categories. The number scored (a) is the total number of male and female embryos.
(DOC)

Table S2 Male lethal mutations of *Sxl* suppress the synthetic female lethality. All females were crossed with *Sxl^f1^/*Y males at 29°C. Female viability was calculated as ((#females)/(#males))100 except in crosses with *Sxl^M^* mutations that affected male viability. In those crosses female viability was calculated as (# females)/(2(non-mutant males)). %*Sxl^+^* surviving is calculated as (# *Sxl^+^* females/# *Sxl^+^* females expected)100. % *Sxl^M^* surviving is calculated as as (# *Sxl^M^* females/# *Sxl^M^* females expected)100. Number scored = total number counted. NA = not applicable.
(DOC)

## References

[1] Merrick WC, Hershey JWB (1996). Translational Control..

[2] Gingras AC, Raught B, Sonenberg N (1999). eIF4 Initiation Factors: Effectors of mRNA Recruitment to Ribosomes and Regulators of Translation.. Annu Rev Biochem.

[3] Sachs AB, Sarnow P, Hentze MW (1997). Starting at the beginning, middle, and end: translation initiation in eukaryotes.. Cell.

[4] Sonenberg N, Gingras AC (1998). The mRNA 5' cap-binding protein eIF4E and control of cell growth.. Curr Opin Cell Biol.

[5] Lejbkowicz F, Goyer C, Darveau A, Neron S, Lemieux R (1992). A fraction of the mRNA 5' cap-binding protein, eukaryotic initiation factor 4E, localizes to the nucleus.. Proc Natl Acad Sci U S A.

[6] Lang V, Zanchin NI, Lunsdorf H, Tuite M, McCarthy JE (1994). Initiation factor eIF-4E of Saccharomyces cerevisiae. Distribution within the cell, binding to mRNA, and consequences of its overproduction.. J Biol Chem.

[7] Dostie J, Lejbkowicz F, Sonenberg N (2000). Nuclear eukaryotic initiation factor 4E (eIF4E) colocalizes with splicing factors in speckles.. J Cell Biol.

[8] Cohen N, Sharma M, Kentsis A, Perez JM, Strudwick S (2001). PML RING suppresses oncogenic transformation by reducing the affinity of eIF4E for mRNA.. EMBO J.

[9] Strudwick S, Borden KL (2002). The emerging roles of translation factor eIF4E in the nucleus.. Differentiation.

[10] Rousseau D, Kaspar R, Rosenwald I, Gehrke L, Sonenberg N (1996). Translation initiation of ornithine decarboxylase and nucleocytoplasmic transport of cyclin D1 mRNA are increased in cells overexpressing eukaryotic initiation factor 4E.. Proc Natl Acad Sci U S A.

[11] Topisirovic I, Capili AD, Borden KL (2002). Gamma interferon and cadmium treatments modulate eukaryotic initiation factor 4E-dependent mRNA transport of cyclin D1 in a PML-dependent manner.. Mol Cell Biol.

[12] Topisirovic I, Culjkovic B, Cohen N, Perez JM, Skrabanek L (2003). The proline-rich homeodomain protein, PRH, is a tissue-specific inhibitor of eIF4E-dependent cyclin D1 mRNA transport and growth.. EMBO J.

[13] Hromas R, Radich J, Collins S (1993). PCR cloning of an orphan homeobox gene (PRH) preferentially expressed in myeloid and liver cells.. Biochem Biophys Res Commun.

[14] Cline TW, Meyer BJ (1996). Vive la difference: males vs females in flies vs worms.. Annu Rev Genet.

[15] Penalva LO, Sanchez L (2003). RNA binding protein sex-lethal (Sxl) and control of Drosophila sex determination and dosage compensation.. Microbiol Mol Biol Rev.

[16] Penn KMJ, Graham P, Schedl P (2004). Alternative Splicing: Regulation of Sex Determination in Drosophila melanogaster: Elsevier Inc.

[17] Keyes LN, Cline TW, Schedl P (1992). The primary sex determination signal of Drosophila acts at the level of transcription.. Cell.

[18] Bell LR, Maine EM, Schedl P, Cline TW (1988). Sex-lethal, a Drosophila sex determination switch gene, exhibits sex-specific RNA splicing and sequence similarity to RNA binding proteins.. Cell.

[19] Samuels ME, Schedl P, Cline TW (1991). The complex set of late transcripts from the Drosophila sex determination gene sex-lethal encodes multiple related polypeptides.. Mol Cell Biol.

[20] Sosnowski BA, Belote JM, McKeown M (1989). Sex-specific alternative splicing of RNA from the transformer gene results from sequence -dependent splice site blockage.. Cell.

[21] Inoue K, Hoshijima K, Sakamoto H, Shimura Y (1990). Binding of the Drosophila sex-lethal gene product to the alternative splice site of transformer primary transcript.. Nature.

[22] Valcarcel J, Singh R, Zamore PD, Green MR (1993). The protein Sex-lethal antagonizes the splicing factor U2AF to regulate alternative splicing of transformer pre-mRNA.. Nature.

[23] Granadino B, Penalva LO, Green MR, Valcarcel J, Sanchez L (1997). Distinct mechanisms of splicing regulation in vivo by the Drosophila protein Sex-lethal.. Proc Natl Acad Sci U S A.

[24] Zhou S, Yang Y, Scott MJ, Pannuti A, Fehr KC (1995). Male-specific lethal 2, a dosage compensation gene of Drosophila, undergoes sex-specific regulation and encodes a protein with a RING finger and a metallothionein-like cysteine cluster.. EMBO J.

[25] Bashaw GJ, Baker BS (1997). The regulation of the Drosophila msl-2 gene reveals a function for Sex-lethal in translational control.. Cell.

[26] Kelley RL, Wang J, Bell L, Kuroda MI (1997). Sex lethal controls dosage compensation in Drosophila by a non-splicing mechanism.. Nature.

[27] Gebauer F, Merendino L, Hentze MW, Valcarcel J (1998). The Drosophila splicing regulator sex-lethal directly inhibits translation of male-specific-lethal 2 mRNA.. RNA.

[28] Gebauer F, Corona DF, Preiss T, Becker PB, Hentze MW (1999). Translational control of dosage compensation in Drosophila by Sex-lethal: cooperative silencing via the 5' and 3' UTRs of msl-2 mRNA is independent of the poly(A) tail.. EMBO J.

[29] Merendino L, Guth S, Bilbao D, Martinez C, Valcarcel J (1999). Inhibition of msl-2 splicing by Sex-lethal reveals interaction between U2AF35 and the 3' splice site AG.. Nature.

[30] Forch P, Merendino L, Martinez C, Valcarcel J (2001). Modulation of msl-2 5' splice site recognition by Sex-lethal.. RNA.

[31] Beckmann K, Grskovic M, Gebauer F, Hentze MW (2005). A dual inhibitory mechanism restricts msl-2 mRNA translation for dosage compensation in Drosophila.. Cell.

[32] Suissa Y, Kalifa Y, Dinur T, Graham P, Deshpande G (2010). Hrp48 attenuates Sxl expression to allow for proper notch expression and signaling in wing development.. PNAS.

[33] Penn JKM, Schedl P (2007). The master switch gene Sex-lethal promotes female development by negatively regulating the N-signaling pathway. Dev.. Cell.

[34] Gebauer F, Gskovic M, Hentze M (2003). *Drosophila* Sex-lethal inhibits the stable association if the 40S ribosomal subunit with msl-2 mRNA.. Mol Cell.

[35] Abraza I, Gebauer F (2005). Functional domains of Drosophila UNR in translational control.. RNA.

[36] Abraza I, Coll O, Patalano S, Gebauer F (2006). Drosophila UNR is required for translational repression of male-specific lethal 2 mRNA during regulation of X-chromosom dosage compensation.. Genes Dev.

[37] Duncan K, Grskovic M, Strein C, Beckmann K, Niggeweg R (2006). Sex-lethal imparts a sex-specific function to UNR by recruiting it to the msl-2 mRNA 3’UTR: translational repression for dosage compensation.. Genes Dev.

[38] Duncan KE, Strein C, Hentze MW (2009). The SXL-UNR co-repressor complex uses a PABP-mediated mechanism to inhibit ribosome recruitment to msl-2 mRNA. Mol.. Cell.

[39] Yanowitz JL, Deshpande G, Calhoun G, Schedl PD (1999). An N-terminal truncation uncouples the sex-transforming and dosage compensation functions of sex-lethal.. Mol Cell Biol.

[40] Harper DS, Fresco LD, Keene JD (1992). RNA binding specificity of a Drosophila snRNP protein that shares sequence homology with mammalian U1-A and U2-B" proteins.. Nucleic Acids Res.

[41] Flickinger TW, Salz HK (1994). The Drosophila sex determination gene snf encodes a nuclear protein with sequence and functional similarity to the mammalian U1A snRNP protein.. Genes Dev.

[42] Polycarpou-Schwarz M, Gunderson SI, Kandels-Lewis S, Seraphin B, Mattaj IW (1996). Drosophila SNF/D25 combines the functions of the two snRNP proteins U1A and U2B' that are encoded separately in human, potato, and yeast.. RNA.

[43] Deshpande G, Samuels ME, Schedl PD (1996). Sex-lethal interacts with splicing factors in vitro and in vivo.. Mol Cell Biol.

[44] Salz HK, Flickinger TW (1996). Both loss-of-function and gain-of-function mutations in snf define a role for snRNP proteins in regulating Sex-lethal pre-mRNA splicing in Drosophila development.. Genetics.

[45] Nagengast AA, Stitzinger SM, Tseng CH, Mount SM, Salz HK (2003). Sex-lethal splicing autoregulation in vivo: interactions between SEX-LETHAL, the U1 snRNP and U2AF underlie male exon skipping.. Development.

[46] Oliver B, Pauli D (1998). Suppression of distinct ovo phenotypes in the Drosophila female germline by maleless- and Sex-lethal.. Dev Genet.

[47] Maine EM, Salz HK, Schedl P, Cline TW (1985). Sex-lethal, a link between sex determination and sexual differentiation in Drosophila melanogaster.. Cold Spring Harb Symp Quant Biol.

[48] Kraut R, Menon K, Zinn K (2001). A gain-of-function screen for genes controlling motor axon guidance and synaptogenesis in Drosophila.. Curr Biol.

[49] Lachance PE, Miron M, Raught B, Sonenberg N, Lasko P (2002). Phosphorylation of Eukaryotic Translation Initiation Factor 4E Is Critical for Growth.. Molec Cell Biol.

[50] Salz HK, Cline TW, Schedl P (1987). Functional changes associated with structural alterations induced by mobilization of a P element inserted in the Sex-lethal gene of Drosophila.. Genetics.

[51] Albrecht EB, Salz HK (1993). The Drosophila sex determination gene snf is utilized for the establishment of the female-specific splicing pattern of Sex-lethal.. Genetics.

[52] Granadino B, Torres M, Bachiller D, Torroja E, Barbero JL (1991). Genetic and molecular analysis of new female-specific lethal mutations at the gene Sxl of Drosophila melanogaster.. Genetics.

[53] Horabin, JI, Schedl P (1996). Splicing of the Drosophila Sex-lethal early transcripts involves exon skipping that is independent of Sex-lethal protein.. RNA.

[54] Zhu C, Urano J, Bell LR (1997). The Sex-lethal early splicing pattern uses a default mechanism dependent on the alternative 5’ splice sites l997 Mol Cell Biol.

[55] Bernstein M, Lersch RA, Subrahmanyan L, Cline TW (1995). Transposon insertions causing constitutive Sex-lethal activity in Drosophila melanogaster affect Sxl sex-specific transcript splicing.. Genetics.

[56] Horabin JI, Schedl P (1993). Sex-lethal autoregulation requires multiple cis-acting elements upstream and downstream of the male exon and appears to depend largely on controlling the use of the male exon 5' splice site.. Mol Cell Biol.

[57] Ding D, Parkhurst SM, Halsell SR, Lipshitz HD (1993). Dynamic Hsp83 RNA localization during Drosophila oogenesis and embryogenesis.. Mol Cell Biol.

[58] Stitzinger SM, Pellicena-Palle A, Albrecht EB, Gajewski KM, Beckingham KM (1999). Mutations in the predicted aspartyl tRNA synthetase of *Drosophila* are lethal and function as dosage sensitive maternal modifiers of the sex determination gene *Sex-Lethal*. Mol. Gen. Genet..

[59] Penalva LO, Ruiz MF, Ortega A, Granadino B, Vicente L (2000). The Drosophila fl(2)d gene, required for female-specific splicing of Sxl and tra pre-mRNAs, encodes a novel nuclear protein with a HQ-rich domain..

[60] Oretga A, Niksic M, Bachi A, Wilm M, Sanchez L (2003). Biochemical function of female lethal 2D/Wilms’ tumor suppressor 1 associated proteins in alternative pre-mRNA splicing.. J Biol Chem.

[61] Penn, JKM, Graham P, Deshpande G, Calhoun G, Chaouki AS (2008). Functioning of the Drosophila Wilms’-Tumor-1-Associated Protein Homolog, Fl(2)d, in Sex-Lethal Dependent Alternative Splicing.. Genetics.

[62] Gonzalaz AN, Lu H, Erickson JW (2008). A shared enhancer controls a temporal switch between promoters during Drosophila primary sex determination.. PNAS.

[63] Samuels ME, Bopp D, Colvin RA, Roscigno RF, Garcia-Blanco MA (1994). RNA binding by Sxl proteins in vitro and in vivo.. Mol Cell Biol.

[64] Das R, Reed R (1999). Resolution of the mammalian E complex and the ATP-dependent spliceosomal complexes on native agarose mini-gels.. RNA.

[65] Das R, Shou S, Reed R (2000). Functional association of U2 snRNP with the ATP independent splicosomal complex E.. Mol Cell.

[66] Kent OA, Ritchie DB, MacMillan AM (2005). Characterization of a U2AF-independent commitment complex (E') in the mammalian spliceosome assembly pathway.. Mol Cell Biol.

[67] Sharma S, Falick AM, Black DL (2005). Polypyrimidine tract binding protein blocks the 5' splice site-dependent assembly of U2AF and the prespliceosomal E complex.. Mol Cell.

[68] Black, DL (2003). Mechanisms of alternative pre-messenger RNA splicing. Ann. Rev. Biochem..

[69] House AE, Lynch KW (2008). Regulation of alternative splicing: more than just the ABCs J Biol Chem.

[70] Jurica MS, Moore MJ (2003). Pre-mRNA splicing: awash in a sea of proteins.. Mol Cell.

[71] Stark H, Luhrmann R (2006). Cryo-electron microscopy of spliceosomal components.. Annu Rev Biophys Biomol Struct.

[72] Donmez G, Hartmuth K, Kastner B, Will CL, Luhrmann R (2007). The 5' end of U2 snRNA is in close proximity to U1 and functional sites of the pre-mRNA in early spliceosomal complexes.. Mol Cell.

[73] Spadaccini R, Reidt U, Dybkov O, Will CFR, Stier G (2006). Biochemical and NMR analyses of an SF3b155-p14-U2AF-RNA interaction network involved in branch point definition during pre-mRNA splicing.. RNA.

[74] Deckert J, Hartmuth K, Boehringer D, Nastaran B, Will CL (2006). Protein composition and electron microscopy structure of affinity-purified human spliceosomal B complexes isolated under physiological conditions.. Mol Cell Biol.

[75] Staley JP, Guthrie C (1998). Mechanical devices of the spliceosome: motors, clocks, springs and things.. Cell.

[76] Makarov EM, Makarova OV, Urlaub H, Gentze M, Will CL (2002). Small nuclear ribonucleoprotein remodeling during catalytic activation of the spliceosome.. Science.

[77] Grandino B, Campuzano S, Sanchez L (1990). The Drosophila melanogaster fl(2)d gene is needed for the female-specific splicing of Sex-lethal RNA.. EMBO J.

[78] Zhang G, Taneja KL, Singer RH, Green MR (1994). Localization of pre-mRNA splicing in mammalian nuclei.. Nature.

[79] Beyer L, Osheim YN (1988). Splice site selection, rate of splicing and alternative splicing on nascent transcripts.. Genes Dev.

[80] Bauren G, Wieslander L (1994). Splicing of Balbiani ring 1 gene pre-mRNA occurs simultaneously with transcription Cell.

[81] Listerman I, Sapra AK, Neugebauer KM (2006). Cotranscriptional coupling of splicing factor recruitment and precursor messenger RNA splicing in mammalian cells.. Nat Struct Mol Biol.

[82] Pandya-Jones A, Black D (2009). Co-transcriptional splicing of constitutive and alternative exons.. RNA.

[83] Singh J, Padgett RA (2009). Rates of in situ transcription and splicing in large human genes.. Nat Struct Mol Biol.

[84] Vargas DY, Shah K, Sinha S, Marras Salvatore AE (2010). Single Molecule Imaging of Transcriptionally Coupled and Uncoupled Splicing.. Cell.

[85] Lallena MJ, Chalmers KJ, Llamazares S, Lamond AI, Valcarcel J (2002). Splicing regulation at the second catalytic step by Sex-lethal involves 3' splice site recognition by SPF45.. Cell.

[86] Harmuth K, Urlaub H, Vornlocher HP, Will CL, Gentzel M (2002). Protein composition of human prespliceosomes isolated by a tobramycin affinity-selection method.. Proc Natl Acad Sci U S A.

[87] Jurica MS, Licklider LJ, Gygi SR, Grigorieff NMooreMJ (2002). Purification and characterization of native spliceosomes suitable for native three dimensional structural analysis.. RNA.

[88] Lim SR, Hertel KJ (2004). Commitment to splice site pairing coincides with A complex formation. Mol.. Cell.

[89] Lindsley DL, Zimm GG (1992). The genome of Drosophila melanogaster..

[90] Bell LR, Horabin JI, Schedl P, Cline TW (1991). Positive autoregulation of sex-lethal by alternative splicing maintains the female determined state in Drosophila.. Cell.

[91] Frohman MA, Dush MK, Martin GR (1988). Rapid production of full-length cDNAs from rare transcripts: amplification using a single gene-specific oligonucleotide primer.. Proc Natl Acad Sci U S A.

[92] Lavoie CA, Lachance PED, Sonenberg N, Lasko P (1996). Alternatively spliced transcripts from the Drosophila eIF4E gene produce two different cap-binding proteins.. J Biol Chem.

[93] Rudner DZ, Kanaar R, Breger KS, Rio D (1998). Interaction between subunits of heterodimeric splicing factor U2AF is essential in vivo.. Mol Cell Biol.

[94] Rudner DZ, Kanaar R, Breger KS, Rio D (1996). Mutations in the small subunit of the Drosophila U2AF splicing factor cause lethality and developmental defects. Proc. Natl. Acad. Sci.. USA.

[95] Deshpande G, Stukey J, Schedl P (1995). scute (sis-b) function in Drosophila sex determination.. Mol Cell Biol.

