# Supplementary figures and images for: The Translation Initiation Factor eIF4E Regulates the Sex-Specific Expression of the Master Switch Gene Sxl in Drosophila melanogaster
Source: PLoS Genet. 2011 Jul 28;7(7):e1002185. doi: 10.1371/journal.pgen.1002185 (PMC3145617; doi:10.1371/journal.pgen.1002185)

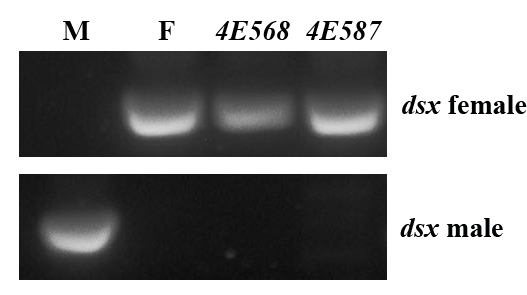

Supplement: Figure S2 — Female specific splicing of dsx mRNA is unaffected by reducing eif4e activity. RNA from wild type males and females and females heterozgyous for either eif4e568 (4E568) or eif4e587/ 11 (4E587) were reverse transcribed with primers specific for the female spliced or male spliced 3′ UTRs. The resulting cDNAs were then PCR amplified using primers complementary to the common exon 3 and the female exon 4 (dsx female), or to the common exon 3 and the first male exon 5 (dsx male). Female specific, but not male specific amplification products are detected in wild type females and in females heterozygous for the two eif4e mutations. To ensure that the amplification products we are seeing are specific dsxF and dsxM, we used nested primers in the common exon for two rounds of PCR amplification. (TIF) [file pgen.1002185.s002.tif]
