# Supplementary material for: The Translation Initiation Factor eIF4E Regulates the Sex-Specific Expression of the Master Switch Gene Sxl in Drosophila melanogaster
Source: PLoS Genet. 2011 Jul 28;7(7):e1002185. doi: 10.1371/journal.pgen.1002185 (PMC3145617; doi:10.1371/journal.pgen.1002185)
Supplement: Table S1 — Sex-lethal staining patterns in older embryos. Unless otherwise indicated females were crossed to Sxlf1 males at 29°C. Progeny were collected as embryos and stained with antibody to Sxl. Embryos at the cellular blastoderm stage or those past nuclear cycle 13 were examined and placed into one of the indicated categories. The number scored (a) is the total number of male and female embryos. (DOC) [file pgen.1002185.s003.doc]

**Supplemental Table 1. Sex-lethal staining patterns in older embryos**

| **Maternal Genotype** | **Staining pattern of**  **late embryos (%)** | | | | **Number scored (a)** |
| --- | --- | --- | --- | --- | --- |
|  | **un-stained** | **even stain** | **patchy stain** | **light stain** |  |
| ***w x w*** | **49** | **49** | **2** | **1** | **313** |
| ***w*** | **49** | **45** | **2** | **3** | **91** |
| ***snf^1621^/+*** | **47** | **11** | **36** | **6** | **260** |
| ***eif4e^587/11^/+*** | **48** | **35** | **11** | **5** | **217** |
| ***eif4e715/+*** | **47** | **30** | **17** | **5** | **264** |
|  | | | | | |
| **Maternal Genotype** | **Staining pattern of**  **early embryos (%)** | | | | **Number Scored** |
|  | **not stained** | | **stained** | |  |
| ***w x w*** | **50** | | **50** | | **59** |
| ***w*** | **40** | | **60** | | **107** |
| ***snf^1621^/+*** | **55** | | **45** | | **106** |
| ***eif4e^587/11^/+*** | **54** | | **46** | | **127** |
| ***eif4e568/+*** | **36** | | **64** | | **86** |
