# Supplementary material for: The Translation Initiation Factor eIF4E Regulates the Sex-Specific Expression of the Master Switch Gene Sxl in Drosophila melanogaster
Source: PLoS Genet. 2011 Jul 28;7(7):e1002185. doi: 10.1371/journal.pgen.1002185 (PMC3145617; doi:10.1371/journal.pgen.1002185)
Supplement: Table S2 — Male lethal mutations of Sxl suppress the synthetic female lethality. All females were crossed with Sxlf1/Y males at 29°C. Female viability was calculated as ((#females)/(#males))100 except in crosses with SxlM mutations that affected male viability. In those crosses female viability was calculated as (# females)/(2(non-mutant males)). %Sxl+ surviving is calculated as (# Sxl+ females/# Sxl+ females expected)100. % SxlM surviving is calculated as as (# SxlM females/# SxlM females expected)100. Number scored = total number counted. NA = not applicable. (DOC) [file pgen.1002185.s004.doc]

**Supplemental Table 2. Male lethal mutations of *Sxl* suppress the synthetic female lethality.**

| **Maternal Genotype** | **Female viability** | **% *Sxl^+^* surviving** | **% *Sxl^M^* surviving** | **Number Scored** |
| --- | --- | --- | --- | --- |
| ***eI^587/11^/+*** | **43** | **NA** | **NA** | **1191** |
| ***Sxl^M1^/+;eif4e******^587/11^/+*** | **64** | **34** | **93** | **618** |
| ***Sxl^M4^/+;eif4e^587/11^/+*** | **63** | **17** | **109** | **186** |
| ***Sxl^M6^/+;eif4e^587/11^/+*** | **67** | **48** | **87** | **769** |
|  |  |  |  |  |
| ***Sxl^M1^/snf^1621^*** | **68** | **10** | **126** | **1773** |
